# Supplementary material for: Vulnerability of progeroid smooth muscle cells to biomechanical forces is mediated by MMP13
Source: Nat Commun. 2020 Aug 17;11:4110. doi: 10.1038/s41467-020-17901-2 (PMC7431909; doi:10.1038/s41467-020-17901-2)
Supplement: Supplementary file 1 — Supplementary Information [file 41467_2020_17901_MOESM1_ESM.pdf]

Supplementary Materials for

Vulnerability of progeroid smooth muscle cells to biomechanical forces is mediated by MMP13

Patricia R. Pitrez[1,2], Luís Estronca[1,2], Luís Monteiro[1], Guillem Colell[3], Helena Vazão[1], Deolinda Santinha[1,2], Karim Harhouri[4], Daniel Thornton[5], Claire Navarro[6], Anne-Laure Egesipe[7], Tânia Carvalho[8], Rodrigo L. dos Santos[9], Nicolas Lévy[4], James C. Smith[10], João Pedro de Magalhães[1,5], Alessandro Ori[11], Andreia Bernardo[10], Annachiara De Sandre-Giovannoli[4], Xavier Nissan[7], Anna Rosell[3], Lino Ferreira[1,2]*

Affiliations:

[1]Center for Neuroscience and Cell Biology, University of Coimbra, Coimbra, Portugal

[2]Faculty of Medicine, University of Coimbra, 3000-548, Coimbra, Portugal

[3]Neurovascular Research Laboratory. Vall d'Hebron Research institute. Passeig Vall d'Hebron 119-129, 08035, Barcelona, Spain

[4]Molecular Genetics Laboratory, Department of Medical Genetics, La Timone Children's hospital, Marseille, France

[5]Integrative Genomics of Ageing Group, Institute of Ageing and Chronic Disease, University of Liverpool, Liverpool L7 8TX, United Kingdom

[6]Aix Marseille Univ, INSERM, GMGF, Marseille, France

[7]CECS, I-STEM, AFM, Institute for Stem Cell Therapy and Exploration of Monogenic diseases, Evry cedex, France

[8]IMM, Instituto de Medicina Molecular, Universidade de Lisboa, Lisbon, Portugal

[9]Cambridge Science Park, Mogrify Ltd, 25 Milton Road, Cambridge, CB4 0FW, UK

[10]Developmental Biology Laboratory, The Francis Crick Institute, London, NW1 1AT UK

[11]Leibniz Institute on Aging-Fritz Lipmann Institute, 07745 Jena, Germany

*Correspondence to: lino@uc-biotech.pt

This PDF file includes:

Supplementary Methods
Supplementary Figures 1-22
Supplementary Tables 1-3
References 1-10

SUPPLEMENTARY METHODS

Cell culture. HGPS Fibroblasts line AG06917 (Coriell cell repositories) was cultured in DMEM (Sigma) supplemented with fetal bovine serum (FBS, 20%, v/v, Gibco), sodium pyruvate (Sigma, 1 mM) and penicillin-streptomycin (50 U/mL:50 mg/mL). hVSMCs (Lonza, CC-2579) were cultured in Smooth Muscle Growth Medium-2 (SmGM-2) medium (Lonza CC-3182) from passage 3 to passage 7. HaCaT (human immortalized keratinocyte cell line) were cultured in DMEM (Sigma) supplemented with FBS (10%, v/v, Gibco) and penicillin-streptomycin (50 U/mL:50 mg/mL). Cell cultures were maintained at 37 ºC, 5 % CO_2 in a humidified atmosphere, with media changed every 2 days.

Characterization of HGPS-iPSC SMCs: intracellular Ca^{2+} variation measurements. Measurements of intracellular Ca^{2+} were performed according to Vazão, H. *et al.* [1]. hVSMCs or HGPS-iPSC SMCs or N-iPSC SMCs were loaded with a Fura-2 calcium fluorescent indicator solution formed by acetoxymethyl (AM) derivative FURA-2/ AM (5 mM, 1 mM in DMSO, Invitrogen), Pluronic F-127 (0.06%, w/v, Sigma) and M199 basal medium (Sigma) (35 µL/well, not supplemented with serum nor antibiotics), for 1 h at 37ºC in 5% CO2 and 90% humidity. Cells were then stimulated with histamine (100 µM, Sigma) or angiotensin (10^{-5} M, Calbiochem), by adding 1 mL of a stock solution. hVSMCs and HGPS Fibroblasts were used as controls.

Characterization of HGPS-iPSC SMCs: contractility assays. Measurements of intracellular Ca^{2+} were performed according to Vazão, H. *et al.* [1]. HGPS-iPSC SMCs and N-iPSC SMCs cultured for 8 passages were washed with DMEM (Sigma) and contraction was induced by incubating these cells with 10^{-5} M carbachol (AlphaAesar) in DMEM (Sigma) medium for 30 min. Contraction was calculated as the difference in cell area (assessed by microscopy)

between time zero and 30 min. In a distinct experiment, cell relaxation was induced by incubation with atropine (10^{-4} M, AlfaAesar) in DMEM (Sigma) for 1 h followed by contraction with carbachol (10^{-5} M, AlphaAesar). Contraction was calculated as mentioned above. hVSMCs and HACAT were used as positive and negative controls, respectively.

Quantitative reverse transcription-polymerase chain reaction (qRT-PCR) analyses. Total RNA was extracted from samples using RNeasy Micro Kit (Qiagen) followed by cDNA synthesis using Taqman Reverse Transcription Reagents for RT-PCR (Thermo Fisher), according to manufacturer’s instructions. Messenger RNA levels from experimental groups were quantified using a Power SYBR® Green Cells-to-CT™ Kit (Applied Biosystems). All genes were measured using SYBR Green technology, with the exception of Progerin. Progerin-specific Taqman primer (Supplementary Table 1) and probe was customized and the results were normalized to glyceraldehyde 3-phosphate dehydrogenase (GAPDH, VIC® / MGB Probe, Primer Limited) (Applied Biosystems). qRT-PCR analyses were performed using an ABI PRISM 7500 Fast System (Applied Biosystems) run for 40 cycles, using 7500 Fast SDS Software v1.4.1. Quantification of target genes was performed relative to GAPDH gene according to the equation: $2^{[-(\text{Ct sample} - \text{Ct GADPH})]}$. The mean minimal cycle threshold values (Ct) were calculated from quadruplicate reactions. The list of the primers can be found in Supplementary Table 2.

Immunofluorescence analysis. Cells were washed with PBS, fixed with 4% paraformaldehyde (Electron Microscopy Sciences) for 15 min at room temperature and washed again with PBS. After fixation, cells were treated with 1% Triton X-100 in PBS for 10 min at RT. Cells were blocked with 1% (w/v) BSA in PBS and stained for 1 h with anti-human primary antibodies specific for smooth muscle α-actin (α-SMA, 1A4, Dako), smooth muscle

myosin heavy chain (1:50 for staining, SMMHC, SMMS-1, Dako), calponin (1:50 for staining, CALP, Calponin1, Santa Cruz Biotec), Lamin A/C (1:50 for staining, H-110, Santa Cruz Biotec), Progerin (1:50 for staining, 13A3DD4, Santa Cruz Biotec), osteopontin (1:50 for staining, AKm2A1, Santa Cruz Biotec), Ki-67 (1:50 for staining, Clone MIB-1, Dako), γH2AX (1:50 for staining, pS139, BD Pharmingen), heparan sulfate (1:50 for staining, 10E4 Epitope, USBiological), Oct3/4 (1:50 for staining, C-10, Santa Cruz Biotec), Sox2 (1:50 for staining, E-4, Santa Cruz Biotec), CD31 (1:50 for staining, M0823, DAKO), p21 (1:50 for staining, F-5, Santa Cruz Biotec), fibronectin (1:50 for staining, EP5, Santa Cruz Biotec), collagen type I (1:50 for staining, ab34710, Abcam). In each immunofluorescence experiment, an isotype matched IgG control was used. Binding of primary antibodies to specific cells was detected with anti-mouse IgG Cy3 (1:100 for staining, Sigma), Alexa Fluor 555 (1:500 for staining, Thermo Fisher) or Alexa Fluor 488 (1:50 for staining, Thermo Fisher), anti-rabbit IgG Cy3 (1:100 for staining, JacksonImmunoResearch). Cell nuclei were stained with 4', 6'-diamidino-2-phenylindole (DAPI) (Sigma) and the slides examined by a high-content fluorescence microscope (IN Cell 2200, GE Healthcare) or a confocal microscope (LSM 710, Zeiss, Germany). ImageJ software or IN Cell developer software were used to quantify the presence or absence of fluoresce and the overall intensity of each image, which was then normalized for cell number.

Metabolic activity. Metabolic activity was assessed by a cell permeable resazurin-based solution, PrestoBlueTM (Life Technologies). PrestoBlueTM Reagent was added directly to cells in culture medium (1:10), incubated for 2 h, and the absorbance’s at 570 and 600 nm monitored by a plate reader (BioTek). The absorbance values at 570 nm were then normalized by the absorbance values at 600 nm.

Caspase-9 activity. Caspase 9, a key initiator of the intrinsic apoptotic pathway of mammalian cells, was measured by a Caspase-Glo® 9 Assay (Promega). Caspase-Glo® 9 Reagent (100 μL) was added to each well of a white-walled 96-well plate containing culture medium (100 μL) without cells (blank) or with cells (sample). The mix was incubated at room temperature for 30 min, after which the luminescence was measured in a plate-reading luminometer (Lumistar). Luminescence values were then normalized by the number of cells per well.

Alkaline phosphatase activity. Alkaline phosphatase activity was assessed either by a colorimetric substrate, 1-Step pNPP (Thermo Fisher), or SigmaFast 5-Bromo-4-chloro-3-indolyl phosphate/Nitro-blue tetrazolium (BCIP/NBT) (Sigma-Aldrich). In the case of 1-Step pNPP substrate, cells were fixed with ethanol 95% (v/v) during 15 min, washed with PBS (Sigma), and finally stained with 1-Step pNPP reagent. The mix was incubated at 37°C for 30 min and the absorbance was monitored at 405 nm using a plate reader (BioTek). Results were normalized by cell number per mm^2. In the case of SigmaFast substrate, cells were fixed with ethanol 95 % (v/v) during 15 min, washed with PBS (Sigma), and then stained with the SigmaFast reagent (one tablet was dissolved in 10 mL of distilled water) for 10-15 min at 37 ºC. Cells were washed three times with distilled water and observed under an optical microscope.

Microarray analyses. The analyses were performed on both HGPS-iPSC SMCs (clone 1) and N-iPSC SMCs at day 0 or day 4 in arterial flow conditions. In both cases, HGPS-iPSC SMCs and N-iPSC SMCs were homogenized in Trizol reagent (Life Technologies) and the total amount of RNA was extracted with RNeasy Micro Kit (Thermo Fisher), according to manufacturer's instructions. RNA quality was assessed by an Agilent 2100 Bioanalyser (G2943CA), using an Agilent RNA 6000 Nano Kit (5067-1511). Gene expression was

evaluated by a whole human genome microarray Human Gene 2.1 ST Array Strip from Affymetrix. The microarrays were scanned by a GeneAtlas Instrument Control v1.0.5 Software from Affymetrix. The raw data were analyzed using Expression Console™ Software from Affymetrix which uses RMA (Robust Multiarray Averaging). Differentially expressed genes were identified also using Affymetrix® Expression Console™ Software.

Statistical analyses of differentially expressed genes and analyses in the context of known ageing and cell senescence genes. The Human Ageing Genomic Resource, in particular the CellAge database containing 279 senescence-associated genes (http://genomics.senescence.info/cells/) was intersected with the results of a differential gene expression analyses between HGPS-iPSC SMCs and N-iPSC SMCs at day 0. Differentially expressed genes were initially identified using the R software at version 3.3.1 and the Limma package (version 3.32) available through Bioconductor (release 3.5). Limma applies a moderated t-test to obtain a differentially expressed list of genes. Genes with a Log2FC >= 1 were overlapped with genes found in the CellAge database. Results were obtained for both the HGPS iPSC SMC panel and the hVSMC panel (Supplementary Data 1), versus normal iPSC SMCs at day 0.

Reactome Pathway analyses were carried out on differentially expressed genes identified from the HGPS iPSC panel at day 4 as compared to day 0 using the Reactome Pathway Analyzer (version 61) and available online (Supplementary Data 2 and Supplementary Data 3). Ingenuity Pathway analysis was further carried out on genes differentially expressed under arterial stress using the following parameters - Molecules per network: defaults; Node types: all; Data sources: all; Confidence: experimentally observed; Species: human; Tissues and cells: all; Mutations: all; Exp. Log Ratio (LogFC) >= 1.6; p-value (adj. p-value) = 0.05.

All microarray data are available at GEO/NCBI (GEO accession: GSE108368, https://www.ncbi.nlm.nih.gov/geo/query/acc.cgi?acc=GSE108368).

Loss of function studies. In case of MMP inhibition studies, a suspension of HGPS-iPSC SMCs (clone 1) or HGPS-iPSC SMCs (clone 2) in SmGM2 medium was seeded in each IBIDI channel. Four hours after seeding, cells were either treated with SmGM-2 medium containing a MMP13 inhibitor (pyrimidine-4,6-dicarboxylic acid, bis-(4-fluoro-3-methyl-benzylamide)) (8 nM, Calbiochem, Merk Millipore), or a broad spectrum MMP inhibitor (20 nM, batimastat, BB-94, Selleckchem). In case of calcification inhibition studies, cells were treated with SmGM-2 medium containing sodium pyrophosphate (0.9 mM, Sigma). In case of farnesyltrasferase inhibitor studies, cells were treated with SmGM-2 medium containing Lonafarnib (20 µM, Absouce Diagnostics). In MMP, calcification and farnesyltransferase inhibition studies, cells were perfused with medium containing the drug at a flow rate of 20 dyne/cm^2. Medium was changed every 7 days. Cell number and viability (Presto Blue assay) was monitored overtime. In case of MMP13 inhibition studies, MMP activity was assessed at day 4, and the percentage of progerin positive cells and the expression of phosphatase alkaline were evaluated at day 7.

In case of siRNA study, lipofectamine RNAiMAX (in DMEM, Life Technologies) was added to siRNA MMP13 (Santa Cruz Biotechnology) or siRNA control (Izasa) in a ratio of 1:1. The complexation of siRNAs with lipofectamine was allowed to proceed for 40 min at room temperature. The complexes were then added to HGPS-iPSC SMCs (clone 2) cultured in SmGM-2 medium in a ratio of 1:3. The culture media was changed after 4 h. The final concentration of siRNA MMP13 was 100 nM.

Morpholino treatment. HGPS-iPSC SMCs were applied to the entry port of an IBIDI channel (µ-Slide VI ^{0,4} Luer, IBIDI). After 18 h, cells were perfused with SmGM-2 medium with PMOs (Ex10 and Ex11 at 20 µM each) at physiological flow rate (20 dyne/cm^2). After 48 h the

medium was changed and a new transfection was performed. Cells without transfection were used as negative control. Cell number was calculated during time to evaluate the detachment of the cells and progerin gene expression was evaluated 96 h after the first transfection of the morpholinos under flow conditions.

Decellularization of the extracellular matrixes. hVSMCs were cultured under flow conditions for 4 days in an IBIDI channel coated with fibronectin (50 µg/mL, Calbiochem). Cells were then washed with PBS and treated with PBS supplemented with ammonium hydroxide (20 mM) and Triton X-100 (0.5 %, v/v) for 5 min at 37 ºC to disrupt lipid-lipid and lipid-protein interactions [2]. The resulting ECM layers were washed with an excess of PBS three times. Then, a suspension of HGPS-iPSC SMCs (clone 1) in SmGM- 2 medium was seeded on top of decellularized ECM and after 4 h, cells were exposed to flow conditions (20 dyne/cm^2).

***Lmna*$^{G609G/G609G}$ SMCs.** Male *Lmna*$^{G609G/G609G}$ and wild-type mice were used [3]. Animal studies were approved by the local ethical committee (Marseille Animal Care Commitee, Protocol n° 96-21122012) and were in accordance with the Directive 2010/63/EU of the European parliament regarding the protection of animals used for experimental and other scientific purposes. Mouse vSMCs (mSMC) were prepared from thoracic aortas of 6 or 18-week old mice. Briefly, after fat tissue removal around aortic region, aorta was dissected from its origin to the proximity of the diaphragm. Aortas from two mice were put into hank's balanced salt solution (HBSS) 1 X, on ice, and then rinsed once in HBSS. Aortas were digested 10 minutes at 37 °C in enzyme solution freshly prepared on the day of isolation (Collagenase 1 mg/ml, Soybean Trypsin inhibitor 1 mg/ml, elastase 0.744 units/ml - Worthington biochemical -, pen/strep 1 %, HBSS 1X). Aortas were then washed off with warmed and equilibrated

DMEM/F12 (20 % FBS inactive, 100 IU/mL penicillin, 100 μg/mL streptomycin). Adventitia was stripped off under the binocular microscope and aortas were opened longitudinally with scissors. ECs layer was removed by gently scrapping the inside of the vessel with forceps. Aortas were placed into a new dish of enzyme solution and incubated at 37 °C for about one or two hours with regular check under microscope regarding cell dissociation. Cells were triturated with a fire polished Pasteur pipette and collected at 200 × g during 5 min, washed twice in DMEM/F12 media and placed in 3 wells of a 48 well dish. After one week, media was replaced. Cells were grown in DMEM/F12 medium that contained 100 IU/mL penicillin, 100 μg/mL streptomycin, and 20 % FBS inactive at 37 °C in a humidified atmosphere at 5 % CO^2. mSMCs were used at passages 4 to 5. Cells were characterized for SMCs and Progeria markers and cultured under flow conditions (120 dyn/cm^2). The loss of mSMCs during time was assessed by the percentage of occupied area.

Histological analyses. For histological analyses, tissue samples from a minimum of 5 mice were collected at the age of 10 weeks. Mice were perfused with PBS and 10 % buffered formalin solution. Subsequently, aortic arches were fixed in 10 % buffered formalin solution for 6 days at room temperature and embedded in paraffin. Paraffin sections (4 µm) were used for immunohistochemistry.

Paraffin tissues sections underwent heat-mediated antigen retrieval using citrate buffer 10 mmol, 0.05% Tween 20 (pH=6) or Tris-EDTA (pH=9) and were blocked with 5 % (w/v) BSA. Subsequently, tissue sections were incubated overnight with primary antibody in 1% BSA. Finally, tissue sections were incubated with secondary antibody, and positive cells were visualized with a high-content fluorescence microscope or Zeiss LSM 710 confocal microscope using a LD Plan-aprochromat 20x/1.0 objective or 40x objective/ 1.4 numerical aperture oil PlanApochromat immersion lens. Antibodies were obtained from the following

sources: smooth muscle α-actin (1:50 for staining, α-SMA, 1A4, Dako), Progerin (1:50 for staining, 13A3DD4, Santa Cruz Biotec) and heparan sulfate (1:50 for staining, 10E4 Epitope, USBiological), Alexa Fluor 555 goat anti-mouse (1:50 for staining, Life Technologies, A21422). Cell nuclei were stained with 4', 6'-diamidino-2-phenylindole (DAPI) (Sigma).

Aortic media thickness. Orcein-stained slides of ascending aorta from each animal were obtained the NanoZoomerSQ (Hamamatsu, Hamamatsu City, Japan). Longitudinal sections of the arteries were analyzed for the following features: (1) media width (from inner elastic lamina do inner adventitial border), and (2) adventitia width. The numerical values for widths of medial and adventitial tunicas were obtained using NDP.view2 software (Hamamatsu, Hamamatsu City, Japan), in sections perpendicular to the aortic axis.

Gene Expression Profiling using Fluidigm. Small parts of mice aortas were frozen to be analyzed. These parts were lysated and homogenized using a MagNA instrument and MagNA Lyser Green Beads (with 5mm (diameter) stainless steel beads). Total RNA was then isolated and quantified. DELTAgene assays (FlexSix - Fluidigm) were designed for human transcripts. The pre-amplification process was performed for 14 cycles in order to obtain sensitivity down to a single cDNA molecule. The oligos were synthesized by Sigma and dissolved at a concentration of 100 μM in water. For each assay a Primer Pair Mix was prepared containing 50 μM Forward Primer and 50 μM Reverse Primer. In order to prepare 10 × Pre-amplification Primer Mix (500 nM each primer), 10 μL of each of the 96 Primer Pair Mixes (50 μM each primer) was mixed with 40 μL buffer consisting of 10 mM Tris–HCl, pH 8.0; 0.1 mM EDTA; 0.25% Tween-20. In order to prepare 10× Assay (5 μM each primer) each Primer Pair Mix was diluted by mixing 10 μL Primer Pair Mix (50 μM each primer) with 90 μL buffer consisting of 10 mM Tris–HCl, pH 8.0; 0.1 mM EDTA; 0.25% Tween-20. A pre-mix containing cDNA and

primers was done and treatment with exonuclease I was performed to remove non-hybridized primers. The Fluidigm® FLEXsix™ Gene expression IFC was used with EvaGreen chemistry. After a prime of the chip, a 10x assay mix and sample mix were prepared and pipetted into the inlets. The chip was loaded, and data was collected using the BioMark HD™. Data was analyzed using Fluidigm® Real Time PCR Analysis v2.1 software. The genes and primer sequences are given in Supplementary Table 3.

Sample preparation for mass spectrometry (mice samples). Pellets from HGPS-iPSC-SMC cells were processed for quantitative proteome analysis using Tandem Mass Tags (TMT), according to Heinze, I. *et al.* [4]. Slides with 4 µm thick sections were deparaffinized in xylene for 2x 5 minutes, rehydrated in 100% ethanol for 2x 5 minutes, and then washed in 96% (v/v), 70% (v/v), 50% (v/v) ethanol and milliQ water for 1x 5 minutes each. Regions of interest were gently scraped using a scalpel and transferred to a PCR tube containing 100 µL of protein solubilization buffer (80 µM Tris pH 8.0, 80 µM DTT and 4% SDS) and processed directly. Samples were sonicated using a Bioruptor Plus (Diagenode) for 25.2 min (15 cycles: 1 min on, 30 sec off) at 20°C using the high setting, and then boiled for 1h at 99°C. Sonication followed by boiling was performed twice. Cysteine residues were alkylated by adding 200 mM iodoacetamide to a final concentration of 15 mM (incubated for 30 min at room temperature in the dark). Reaction was quenched by addition of 10 µL of 200 mM DTT.

The lysates were treated with 4 volumes ice cold acetone and left overnight at -20 °C to precipitate the proteins. The samples were then centrifuged at 20800x g for 30 minutes, 4 °C. After removal of the supernatant, the precipitates were washed twice with 400 µL of a solution of ice cold 80 % acetone (20 % water). After addition of each wash solution, the samples were vortexed and centrifuged again for 10 minutes at 4°C. The pellets were then allowed to air-dry before being dissolved in digestion buffer at 1 µg/µL (3M urea in 0.1M HEPES, pH 8). To

facilitate the resuspension of the protein pellet, the samples were subjected to 3 rounds of sonication in the Bioruptor, as described above. A 1:100 w/w amount of LysC (Wako sequencing grade) was added to each sample and then they were incubated for 4 h at 37 °C with shaking (1000 rpm). The samples were diluted 1:1 with milliQ water (to reach 1.5M urea) and were incubated with a 1:100 w/w amount of trypsin (Promega sequencing grade) overnight at 37 °C, 650 rpm. The digests were then acidified with 10% trifluoroacetic acid and then desalted with Waters Oasis® HLB µElution Plate 30µm in the presence of a slow vacuum. In this process, the columns were conditioned with 3x100 µL solvent B (80% acetonitrile; 0.05% formic acid) and equilibrated with 3x 100 µL solvent A (0.05% formic acid in milliQ water). The samples were loaded, washed 3 times with 100 µL solvent A, and then eluted into PCR tubes with 50 µL solvent B. The eluates were dried down with the speed vacuum centrifuge and dissolved in 5% acetonitrile, 95% milliQ water, with 0.1% formic acid at a concentration of 1µg/µL. 10 µL was transferred to an MS vial and 0.25 µL of HRM kit peptides (Biognosys, Zurich, Switzerland) was spiked into each sample prior to analysis by LC-MS/MS.

Label-free analysis using data-independent acquisition (DIA). Peptides were separated using the nanoAcquity UPLC MClass system (Waters) fitted with a trapping (nanoAcquity Symmetry C18, 5µm, 180 µm x 20 mm) and an analytical column (nanoAcquity BEH C18, 1.7µm, 75µm x 250mm). The outlet of the analytical column was coupled directly to Q-Exactive HFX (Thermo Fisher Scientific) using the Proxeon nanospray source. Solvent A was water, 0.1 % formic acid and solvent B was acetonitrile, 0.1 % formic acid. The samples (approx. 1 µg) were loaded with a constant flow of solvent A at 5 µL/min onto the trapping column. Trapping time was 6 minutes. Peptides were eluted via the analytical column with a constant flow of 0.3 µL/min. During the elution step, the percentage of solvent B increased in a non-linear fashion from 0 % to 40 % in 120 minutes. Total runtime was 145 minutes,

including clean-up and column re-equilibration. The peptides were introduced into the mass spectrometer via a Pico-Tip Emitter 360 µm OD x 20 µm ID; 10 µm tip (New Objective) and a spray voltage of 2.2 kV was applied. The capillary temperature was set at 300 °C. The RF ion funnel was set to 40%. Data from pools of each condition were first acquired in DDA mode to contribute to a sample specific spectral library. The conditions were as follows: Full scan MS spectra with mass range 350-1650 m/z were acquired in profile mode in the Orbitrap with resolution of 60000. The filling time was set at maximum of 20 ms with limitation of 3 x 106 ions. The "Top N" method was employed to take the 15 most intense precursor ions (with an intensity threshold of 4 x 10^4) from the full scan MS for fragmentation (using HCD normalized collision energy, 27%) and quadrupole isolation (1.6 Da window) and measurement in the Orbitrap (resolution 15000, fixed first mass 120 m/z). The peptide match 'preferred' option was selected, and the fragmentation was performed after accumulation of 2 x 10^5 ions or after filling time of 25 ms for each precursor ion (whichever occurred first). MS/MS data were acquired in profile mode. Only multiply charged (2+ - 5+) precursor ions were selected for MS/MS. Dynamic exclusion was employed with maximum retention period of 20 s and relative mass window of 10 ppm. Isotopes were excluded. In order to improve the mass accuracy, internal lock mass correction using a background ion (m/z 445.12003) was applied. For data acquisition and processing of the raw data Xcalibur 4.0 (Thermo Scientific) and Tune version 2.9 were employed.

For the DIA data acquisition the same gradient conditions were applied to the LC as for the DDA and the MS conditions were varied as described: Full scan MS spectra with mass range 350-1650 m/z were acquired in profile mode in the Orbitrap with resolution of 120000. The default charge state was set to 3+. The filling time was set at maximum of 60 ms with limitation of 3 x 10^6 ions. DIA scans were acquired with 34 mass window segments of differing widths across the MS1 mass range. HCD fragmentation (stepped normalized collision energy; 25.5,

27, 30%) was applied and MS/MS spectra were acquired with a resolution of 30000 with a fixed first mass of 200 m/z after accumulation of 3 x 10^6 ions or after filling time of 40 ms (whichever occurred first). Data were acquired in profile mode.

Data analysis. For library creation, the DDA and DIA data were searched independently using Pulsar in Spectronaut Professional+ (version 11.0.15038, Biognosys AG, Schlieren, Switzerland). The data was searched against the mouse Uniprot database (Swissprot entry only, release 2016_01, 16,747 entries) with a list of common contaminants appended. The data was searched with the following modifications: Carbamidomethyl (C) (Fixed), and Oxidation (M) and Acetyl (Protein N-term) (Variable). A maximum of 2 missed cleavages for trypsin were allowed. The identifications were filtered to satisfy FDR of 1 % on peptide and protein level. A DpD (DDA plus DIA) library was then created by merging the two libraries together in Spectronaut. This library contained 32302 precursors, corresponding to 2262 protein groups using Spectronaut protein inference. Differential protein expression was evaluated using a pairwise t-test performed at the precursor level followed by multiple testing correction according to [5] . The data (candidate table) was exported from Spectronaut and used for further data analyses.

The mass spectrometry proteomics data have been deposited to the ProteomeXchange Consortium via the PRIDE [6] partner repository with the dataset identifier PXD011652 (http://proteomecentral.proteomexchange.org/cgi/GetDataset?ID=PXD011652).

Sample preparation for mass spectrometry (HGPS-iPSC-SMC). Pellets from HGPS-iPSC-SMC cells were processed for quantitative proteome analysis using Tandem Mass Tags (TMT). Briefly, samples were lysed with 100 µL of 2% SDS, 50 mM DTT, 100 mM HEPES, pH 8.0 using a Bioruptor Plus (Diagenode). Following reduction and alkylation, proteins were

precipitated with ice cold acetone. Protein pellets were reconstituted in digestion buffer containing 3M urea in 0.1M HEPES, pH 8 and LysC (1:100) (Wako). After 4 h of incubation at 37°C with shaking at 650 rpm, the samples were diluted 1:1 with milliQ water (to reach 1.5M urea) and were incubated with trypsin (1:100) for 16 h at 37°C. Digested peptides were desalted using Waters Oasis® HLB µElution Plate 30µm, dried down using a vacuum centrifuge and labelled using TMT-10plex reagents (Thermo Fisher Scientific). Following verification of labelling efficiency, samples were pooled and fractionated by high pH reverse chromatography using an Agilent 1260 Infinity HPLC System. Forty-eight fractions were collected along the chromatographic separation, which were subsequently pooled into 16 fractions. Pooled fractions were dried and then stored at -80°C until LC-MS/MS analysis. Each fraction was analyzed in an Orbitrap Fusion Lumos (Thermo Fisher Scientific) online connected with a nanoAcquity UPLC system (Waters) using a Synchronus Precursor Selection (SPS)-MS3 method [7].

TMT-10plex data were processed using Proteome Discoverer v2.0 (Thermo Fisher). Data was searched against a human database (Uniprot database, Swissprot entry only, release 2016_01) using Mascot v2.5.1 (Matrix Science) with the following settings: enzyme was set to trypsin, with up to 1 missed cleavage. MS1 mass tolerance was set to 10 ppm and MS2 to 0.5 Da. Carbamidomethyl cysteine was set as a fixed modification and oxidation of Methionine as variable. Other modifications included the TMT-10plex modification from the quantification method used. The quantification method was set for reporter ions quantification with HCD and MS3 (mass tolerance, 20 ppm). The false discovery rate for peptide-spectrum matches (PSMs) was set to 0.01 using Percolator [8].

Reporter ion intensity values for the PSMs were exported and processed with procedures written in R (version 3.4.1). PSMs mapping to reverse or contaminant hits, or having a Mascot score below 15, or having reporter ion intensities below 1 x 103 in all the relevant TMT

channels were discarded. TMT channels intensities from the retained PSMs were then log2 transformed, normalized and summarized into protein group quantities by taking the median value. At least two unique peptides per protein were required for the identification and only those peptides with one missing values across all 10 channels were considered for quantification. Protein differential expression was evaluated using the limma package [9]. Differences in protein abundances were statistically determined using the Student's t test moderated by the empirical Bayes method. *p* values were adjusted for multiple testing using the Benjamini-Hochberg method (FDR, denoted as "adj p") [10]. The mass spectrometry proteomics (Tandem Mass Tags - TMT) data have been deposited to the ProteomeXchange Consortium via the PRIDE partner repository with the dataset identifier PXD019316 (http://proteomecentral.proteomexchange.org/cgi/GetDataset?ID=PXD019316).

Plasmids and sgRNA. The sgRNA used (5'-AGGAGATGGGTCCACCCACC-3') was designed to target the HGPS mutation site, specifically on the mutated HGPS allele. This sgRNA was cloned into a pSpCas9(BB)-2A-GFP (px458) plasmid (Addgene), which contains an insert for Cas9 from *Streptococcus pyogenes*.

Genome editing procedure. Prior to transfection, HGPS iPSCs were incubated for two hours with ROCK inhibitor (5 µM, Tocris), then detached by means of TrypLE Express® (ThermoFischer) and centrifuged for 5 min at 300 × g. Thereafter, 1×10^6 live HGPS iPSCs were resuspended in a 100 µL-reaction volume containing 82 µL of Nucleofector™ solution and 18 µL of Supplement from the Amaxa™ P3 Primary Cell 4D-Nucleofector™ X Kit (Lonza) with 4 µg of sgRNA plasmid (1.7 µg/ µL). The mixture was added into a Nucleocuvette™ (Lonza) and nucleofected using the Nucleofector™ system (Lonza). Nucleofected cells were plated on feeders (CF1 MEFs, generated in house) in KSR medium

supplemented with ROCK inhibitor (5 µM, Tocris). Nucleofected cells were plated into a full 6-well plate to allow for single cell colonies to emerge. After two weeks, 40 single colonies were manually picked and plated in individual 96-well plate wells pre-seeded with CF1 MEFs and in KSR Medium. Individual clones were maintained in culture until genotyping was completed.

Clone genotyping. To isolate genomic DNA from the individual clones, cells from confluent 96-well plate wells were detached using TrypLE Express® (ThermoFischer), spun down (3 min @ 300g), and then resuspended and incubated for 55 min @ 37ºC in 20 µL of Lysis Buffer (lysis buffer composition: 670 mM Tris HCl at pH 9.1, 160 mM $(NH_4)_2SO_4$, 35 mM $MgCl_2$, 1.5 mg/mL BSA; 1 µL of 10% Igepal (Sigma); 0.8 µL of 20 mg/mL Proteinase K (New England Biolabs) and 16.2 µL of dH_2O). Lysis reaction was stopped by incubating the samples for 15 min @ 95ºC. The resulting lysate was used directly in the PCR reaction.

A 1098bp region containing the HGPS mutation was amplified by PCR using a Q5® High-Fidelity DNA Polymerase (New England Biolabs) and pre-tested primers (Amplification Forward primer: 5´-TCTCTCCCCCATTCTTGTTGCA -3'; Amplification Reverse primer: 5´-CATGAGGTGAGGAGGACGCAG-3'). Sanger sequencing was then used to determine if the targeted site had been mutated. To this end, we designed primers for a 500 bp region contained within the 1098 bp amplicons; both forward and reverse primers were used to confirm if the region had been targeted (Sequencing Forward primer: 5´- CCTTGGGCACAGAACCACAC -3'; Sequencing Reverse primer: 5´- GCGGAAGAGAAGGCAGGCTC-3').

DNA extraction and genetic background of different cell types. DNA was extracted from samples HGPS Fibroblasts, HGPS-iPSCs, N-iPSCs and hVSMCs, by the Biological Resources Center (with NF-S 86-900 and ISO-9001 V2015 certifications) of the Department of Medical

Genetics, La Timone Hospital, Marseille, France. Primers were designed for PCR amplification using Primer3 software (http://bioinfo.ut.ee/primer3/) in order to amplify the gene's exon. Briefly, Sanger sequencing was performed as follows: PCR products were purified by mixing with a volume ratio (1/8) (36 µL) of AMPure beads (Beckman Coulter, USA) according to the manufacturer's instructions and both strands were sequenced using the Big Dye Terminator V.1.1 Cycle Sequencing Kit (Applied Biosystems). Sequence reactions were purified on Sephadex G50 (Amersham Pharmacia Biotech, Foster City, California, USA) and capillary electrophoresis was performed on Genetic Analyser ABI3500XL (Life Technologies, USA). Electrophoregrams were analyzed on the Sequence Analysis Software V.5.2 (Applied Biosystems) and aligned and validated relative to the reference sequence using Sequencher V.5.4.6.

Western Blot. Total cell proteins were extracted in 200 µL of NP40 Cell Lysis buffer (Thermo Fisher Scientific Inc. Waltham, MA, USA) containing Protease and Phosphatase Inhibitor Cocktail (Thermo Fisher Scientific). Cells were sonicated 3 times (30 s each), incubated at 4°C for 30 min, vortexed every 10 min and then centrifuged at 10,000 g for 10 min at 4°C. Protein concentration was evaluated with the bicinchoninic acid technique (Pierce BCA Protein Assay Kit), absorbance at 562 nm was measured using nanodrop 1000 (Thermo Fisher Scientific) Equal amounts of proteins (40 µg) were loaded onto 10% Tis-Glycine gel (CriterionTM XT precast gel) using XT Tricine running Buffer (Biorad, Hercules, CA, USA). After electrophoresis, gels were electro transferred onto Immobilon-FL polyvinylidene fluoride membranes (Millipore), blocked in Odyssey Blocking Buffer for 1 h at room temperature, and incubated overnight at 4°C with various primary antibodies diluted in blocking buffer added with 0.1% Tween 20: 1:1000 rabbit polyclonal anti-Lamin A/C (sc-20681, Santa Cruz Biotechnology, Dallas, TX, USA), 1:1000 mouse monoclonal anti-Progerin (ab66587, Abcam,

Cambridge, UK), 1:5000 mouse monoclonal anti-actin (MAB1501, Merck Millipore, Darmstadt, Germany) and 1:1000 monoclonal anti-glyceraldehyde-3-phosphate dehydrogenase (GAPDH) (MAB374, Merck Millipore). Blots were washed with TBS-T buffer 3 x 10 minutes [20 mM tris (pH 7.4), 150 mm NaCl, and 0.05% Tween 20] and incubated with 1:10,000 IR-Dye 800-conjugated secondary donkey anti-rabbit or IR-Dye 700-conjugated secondary anti-mouse antibodies (LI-COR Biosciences) in blocking buffer added with 0.1% Tween 20 (LI-COR Biosciences, Lincoln, NE, USA). Blots were washed with TBS-T 3x10 minutes. For IR-Dye 800 and IR-Dye 700 detection, an Odyssey Infrared Imaging System (LI-COR Biosciences) was used. GAPDH and actin were used as a total cellular protein loading control.

Validation of MMP13 knockout mice. Genomic DNA was extracted from tail tips using the PureLink® Genomic DNA kit (Thermofisher) according to a protocol provided by the manufacturer. Polymerase chain reaction was performed using Platinum® Taq DNA polymerase (Life Technologies, USA). The following oligonucleotide primers were used to genotype the wild-type mice and knockout mice: (Exon 5F) 5′TTTATTGTTGCTGCCCATGAG3′, (Exon 6R) 5′AGTTTCTCCTCGGAGACTGGT3′ and a primer for the neomycin resistance gene, (neo) 5′GACCCA CCCCTTCCCAGCCTCT3′ was used. The approximate fragment sizes of each band were 1300 Kb for the wild-type and 1485 Kb for the MMP-13 KO on agarose gels. For *Lmna* genotyping the following oligonucleotide primers were used: *Lmna* #1: 5’AAGGGGCTGGGAGGACAGAG3’, *Lmna* #2: 5’AGTAGAAGGTGGCGCGAAGG3’ and *Lmna* #3: 5’AGCATGCAATAGGGTGGAAGGA3’. The approximate fragment sizes of each band for wild-type *Lmna* allele was 100 bp and for the Excised G609G allele was 240 bp on agarose gels. Control samples from known genotypes and bp markers were run in all agarose gels.

Karyotype Analysis. The karyotype of HGPS-iPSCs and HGPSΔ2-iPSC samples were analyzed using KaryoStat™ assay (ThermoFisher Scientific). Briefly, genomic DNA (gDNA) was extracted using PureLink Genomic DNA Mini Kit (catalog # K182000) and estimated by Qubit dsDNA BR Assay Kits. gDNA was processed according to manufacturer's instructions. Briefly, gDNA was digested with Nsp I restriction enzyme. Digested DNA was adapter ligated and PCR amplified. DNA was then fragmented and labeled with biotin. Fragmented-labeled DNA was hybridized onto GeneChip arrays in a GeneChip Hybridization Oven 645 overnight. Chips were washed and stained simultaneously using GeneChip Fluidics Station 450 and scanned using GeneChip Scanner 3000 7 G. Data were analyzed using ChAS 3.2. The whole genome view displays all somatic and sex chromosomes in one frame with high level copy number. The smooth signal plot (right y-axis) is the smoothing of the log2 ratios which depict the signal intensities of probes on the microarray. A value of 2 represents a normal copy number state (CN = 2). A value of 3 represents chromosomal gain (CN = 3). A value of 1 represents a chromosomal loss (CN = 1). The pink, green and yellow colors indicate the raw signal for each individual chromosome probe, while the blue signal represents the normalized probe signal which is used to identify copy number and aberrations (if any).

Flow cytometry analyses. HUAECs were cultured for 3 days in EGM2 (Lonza) or SmGM2 (Lonza) media. Then, cells were dissociated with non-enzymatic cell dissociation buffer (Gibco) for 10 min, followed by gentle pipetting and washes in PBS with 5% (v/v) FBS. Single cells were aliquoted in PBS with 5% FBS (200,000 cells were used per condition) and stained with either isotype controls or antigen-specific fluorescent-conjugated antibodies (CD34-APC, 130-090-954 MACS Miltenyi Biotec, 1:20 dilution) for 30 min at 4ºC. For VE-Cadherin (F-8, sc-9989, Santa Cruz Biotech, 1:20 dilution) and KDR (anti-VEGF receptor 2 antibody

[EPR21231] (ab234110, Abcam, 1:20 dilution) analysis cells were fixed with 1% paraformaldehyde (PFA) for 10 min, permeabilized with triton 0.1% for 10 min, and were incubated with primary antibody for 1 h at room temperature followed by the secondary antibody for 30 min at room temperature. The flow cytometry analyses were performed in a BD Accuri C6 and processed with FlowJo_V10. At least ten thousand events were collected. The percentages showed in the histograms were calculated based on the isotype controls represented by orange (the gate for the isotype was defined at 1%).

Co-culture studies. A suspension of HGPS-iPSC SMCs or N-iPSC SMCs (8.33×10^4 cells/cm^2) was applied to the entry port of an IBIDI channel (μ-Slide VI ^{0,4} Luer, IBIDI) and allowed to flow inside by capillary force. A confluent cell layer was formed after 4 h after which HUAECs were seeded on top of the HGPS-iPSC SMCs or N-iPSC SMCs. Three different densities of HUAECs were applied, 5×10^4 cells/cm^2; 8.33×10^4 cells/cm^2 and 1.3×10^4 cells/cm^2 to get 3 different EC:SMC ratio´s, i.e., 0.6, 1 and 1.6, respectively. After 4 h, the co-culture of cells was then perfused with EGM-2 medium at physiological flow rate (20 dyne/cm^2). All tests were performed at days 0 and 6. Immunofluorescence analyses for HUAECs and SMCs were performed using antibodies for CD31 and calponin, respectively. IBIDI channels were examined by a high-content fluorescence microscope (IN Cell 2200, GE Healthcare) or a confocal microscope (LSM 710, Zeiss, Germany). ImageJ software or IN Cell developer software were used to quantify cell number for each cell type which was then normalized for day 0.

Senescence associated β-galactosidase (SA-βgal) activity. The assay to detect SA-βgal was based on the hydrolysis of a membrane permeable molecule, the 5-dodecanoylaminofluorescein di-β-D-galactopyranoside (C12FDG). Briefly, HGPS-iPSC

SMCs and N-iPSC SMCs were in 96-well plate, and their culture media was removed and replaced by pre-warmed fresh culture media. Cells were treated with a final concentration of 100 nM of bafilomycin A1 (Sigma, in 20 µM DMSO), which was used to neutralize the acidic pH of lysosomes. Cells were incubated for 1 h at 37 °C, 5% CO_2. Then, C12FDG was added to cells at a final concentration of 33 µM (in 20 mM DMSO) and incubate 2 h at 37 °C, 5% CO_2. Finally, cell nuclei were stained with Hoechst 33342 (Molecular Probes®) and cells examined by a high-content fluorescence microscope (IN Cell 2200, GE Healthcare). IN Cell developer software was used to quantify the presence or absence of fluoresce using a threshold based, which was then normalized for cell number.

Quantification of total Pro-MMP13 and endogenous active form of MMP13. Cell culture media collected from HGPS-iPSC SMCs and N-iPSC SMCs, cultured or not 4 days under flow conditions (day 0 and day 4) were used for MMP13 quantification based in a commercial kit, i.e., SensoLyte ® Plus 520 MMP-13 Assay Kit Fluo (089AS-72019, AnaSpec). For each technical replicate, of each experimental group, we divided the cell culture media in two wells. In one of the wells (to calculate the endogenous active form of MMP13), we added a MMP substrate (5-FAM/QXL™ peptide 520 substrate, 100 µL) to the cell culture media and the fluorescence intensity measured in a fluorimeter (Ex/Em=490/520 nm) after 1 h, at room temperature. In the second well (to calculate the total Pro-MMP13 form), we have activated all the MMP13 in the cell culture media by adding 4-aminophenylmercuric acetate (APMA, 100 µL, 1 mM) and incubated for 40 min at 37ºC. Then, a MMP substrate (5-FAM/QXL™ peptide 520 substrate, 100 µL) was added to the mixture and the fluorescence intensity measured in a fluorimeter (Ex/Em=490/520 nm) after 1 h, at room temperature. Concentration of total Pro-MMP13 and endogenous active form of MMP13 was then calculated and normalized by cell number.

Overexpression of MMP13. MMP13 lentiviral particles, Lenti ORF particles, MMP13 (RC224156L1V, Origene Technologies Inc) were used to induce the expression of MMP13 in hVSMCs. A suspension of hVSMCs (P5, 1.11×10^4 cells/cm^2) was seeded in 24 well plates to obtain 50% confluence after 1 day. Then, hVSMCs were infected with lentiviral particles (2.5 μL, 6.3×10^7 TU / mL) in 250 μL of SmGM2. More medium (250 μL) was added in the next day. Day 3 post-infection cells were analyzed by qRT-PCR and cultured under flow conditions for 7 days.

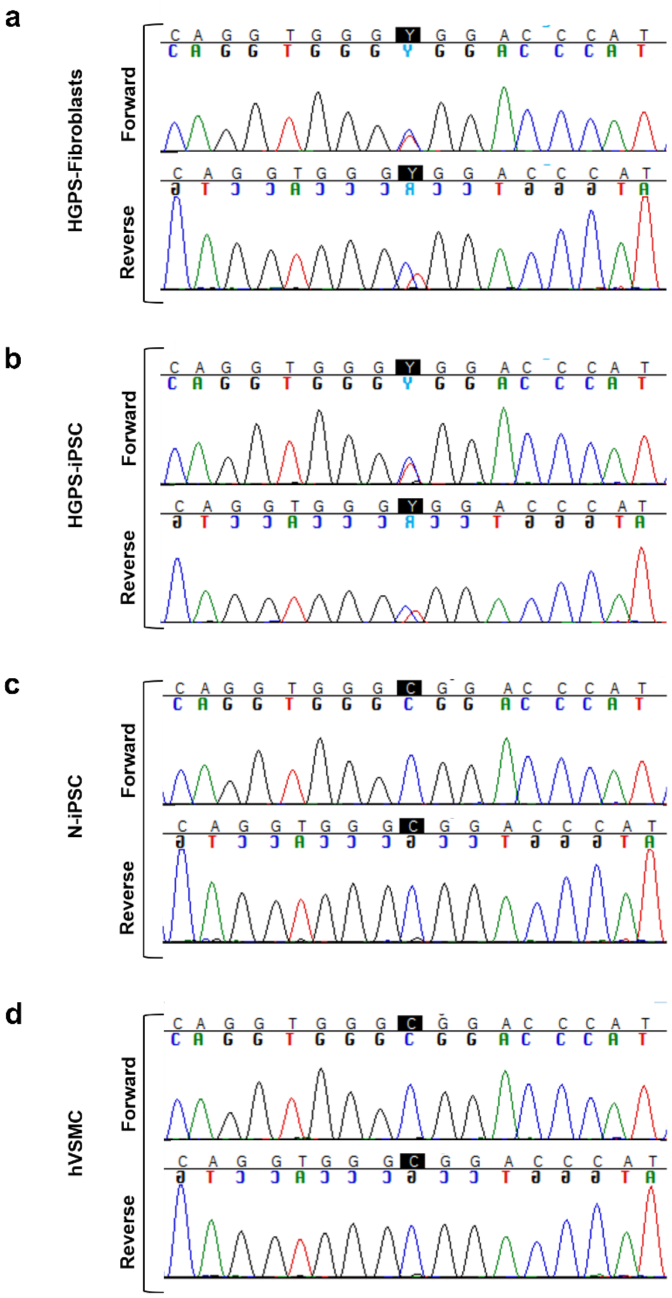

Supplementary Figure 1: Genetic background of N-iPSCs, HGPS-iPSCs, HGPS Fibroblasts and hVSMCs. Sanger sequencing for *LMNA* (NM_170707.4 transcript) exon 11 was performed for**:** **a** HGPS Fibroblasts, **b** HGPS-iPSCs, **c** N-iPSCs and **d** hVSMCs.

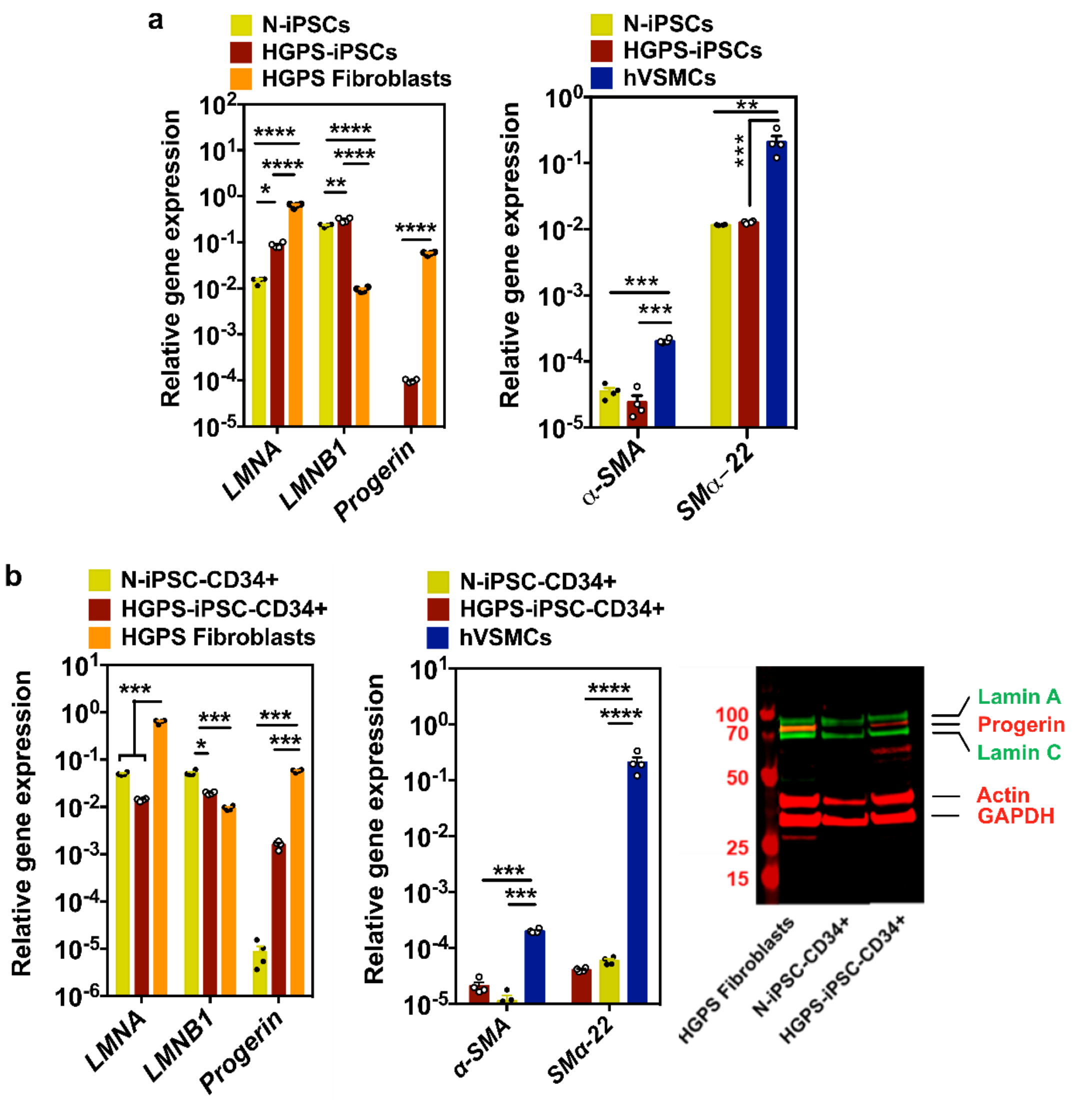

Supplementary Figure 2: Expression of progeria and SMC markers in undifferentiated iPSCs and CD34+ cells. a Expression of progeria (progerin and lamins) and SMC (*α-SMA*, *SMα-22*) markers on undifferentiated HGPS-iPSCs and N-iPSCs, as evaluated by qRT-PCR analyses. HGPS Fibroblasts and hVSMCs were used as controls. **b** Characterization of CD34+ cells at passage 1. Gene expression of lamins (*lamin A*, *lamin B1*), progerin and SMC markers. Progerin and lamin A/C protein expression were also analyzed by western blot. In **a**, **b,** results are Mean ± SEM (n=4 technical replicates from a pool of 3 independent experiments, except for western blot, n=2 independent experiments). *,**,***,**** denotes statistical significance (p<0.05, p<0.01, p<0.001, p <0.0001). Gene expression was normalized by the expression of housekeeping gene *GAPDH*. N-iPSCs refers to iPSCs without the disease state; HGPS-iPSCs refers to iPSCs with Progeria; and hVSMCs refers to normal human vascular SMCs. From **a** to **b**, statistical analyses were performed by one-way ANOVA followed by Newman Keuls's post-test.

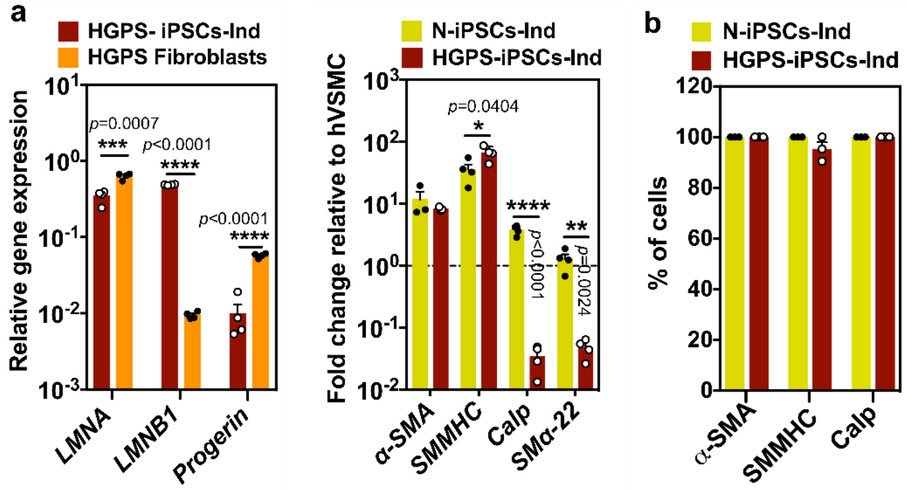

Supplementary Figure 3: Expression of progeria and SMC markers after 4 passages using SMC inductive media. a Expression of lamins (*lamin A, lamin B1), progerin* and SMC gene markers in cells differentiated from N- or HGPS-iPSCs for 4 passages (ca. 15 days) in inductive media. Gene expression was normalized by the expression of housekeeping gene *GAPDH*. Results are Mean ± SEM (n=4 technical replicates from a pool of 3 independent experiments). *,***,**** denotes statistical significance (p<0.05, p<0.001, p<0.0001). In case of SMC gene expression, the values were normalized by the corresponding gene expression in hVSMCS. **b** Percentage of cells that express SMC proteins at the inductive stage of cell differentiation. Results are Mean ± SEM (n=3 independent experiments). Our results show that cells (HGPS-iPSCs- Ind or N-iPSCs-Ind) after culture in SMC inductive media express high levels of mRNA progerin as well as SMC transcripts and proteins. Statistical analyses were performed by two-tailed unpaired Student's t-test.

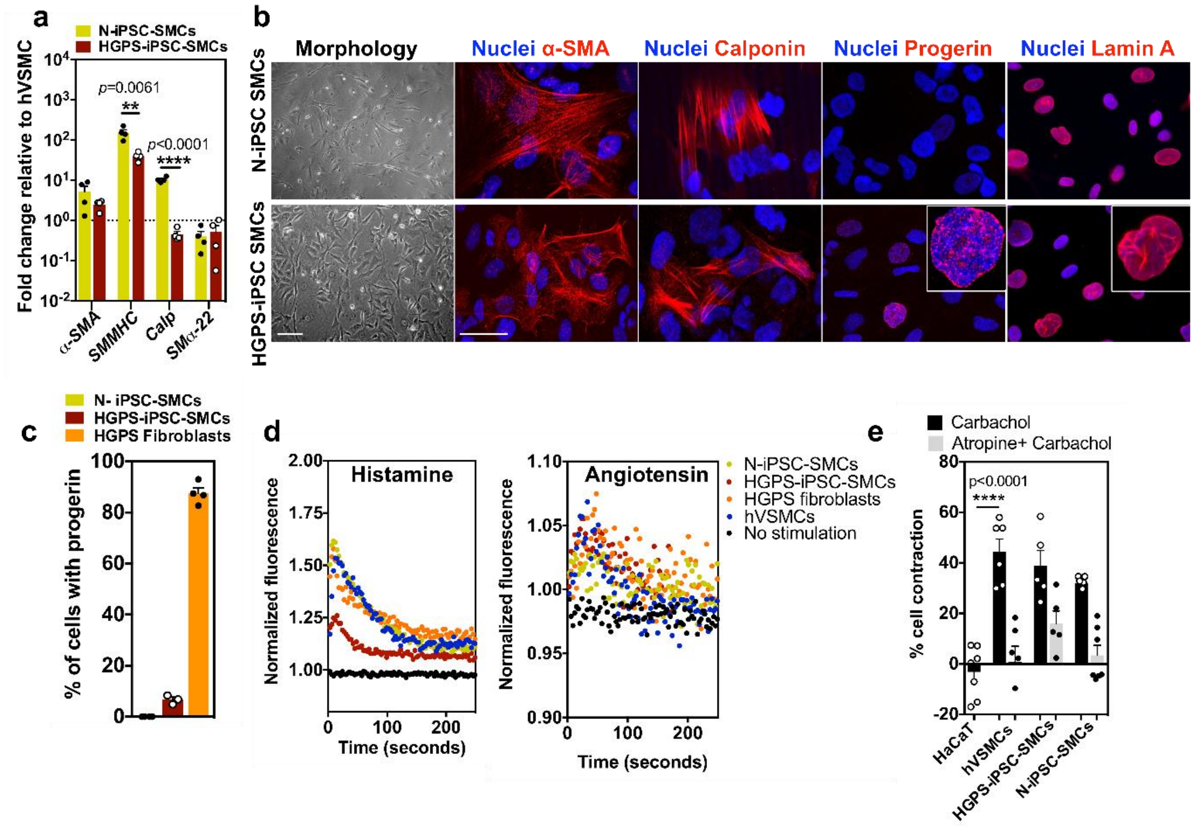

Supplementary Figure 4: Derivation and characterization of SMCs from N-iPSCs and HGPS-iPSCs. a Gene expression by qRT-PCR. The expression of each gene was normalized by the expression of *GAPDH* and then by the gene expression observed in hVSMCs. Results are Mean ± SEM (n=4 technical replicates from a pool of 3 independent experiments). **,*** denotes statistical significance ($p<0.01$, $p<0.001$). Statistical analyses were performed by two-tailed unpaired Student's t-test. **b** Immunofluorescence analysis performed on HGPS-iPSC SMCs and N-iPSC SMCs at passage 8 for SMC and progerin markers. Scale bar is 50 µm. n=3 independent experiments. **c** Percentage of cells positive for progerin as assessed by immunofluorescence. Although HGPS-iPSC SMCs from both individuals express lower levels of progerin protein than HGPS Fibroblasts, it should be noted that differentiated HGPS-iPSCs were cultured for 8 passages while HGPS Fibroblasts were cultured for more than 24 passages. Results are Mean ± SEM (n=3 independent experiments). **d** Intracellular Ca2+ measurements in cells loaded with FURA-2 and exposed to histamine or angiotensin. hVSMCs, HaCaT and HGPS Fibroblasts were used as controls. **e** Contractility of NiPSC SMCs and HGPS-iPSC SMCs after exposure to carbachol or atropine plus carbachol. hVSMCs and HaCAT were used as controls. Results are Mean ± SEM (n=5-7 technical replicates). **** denotes statistical significance ($p<0.0001$) compared to hVSMCs. Statistical analyses were performed by two-tailed unpaired Student's t-test.

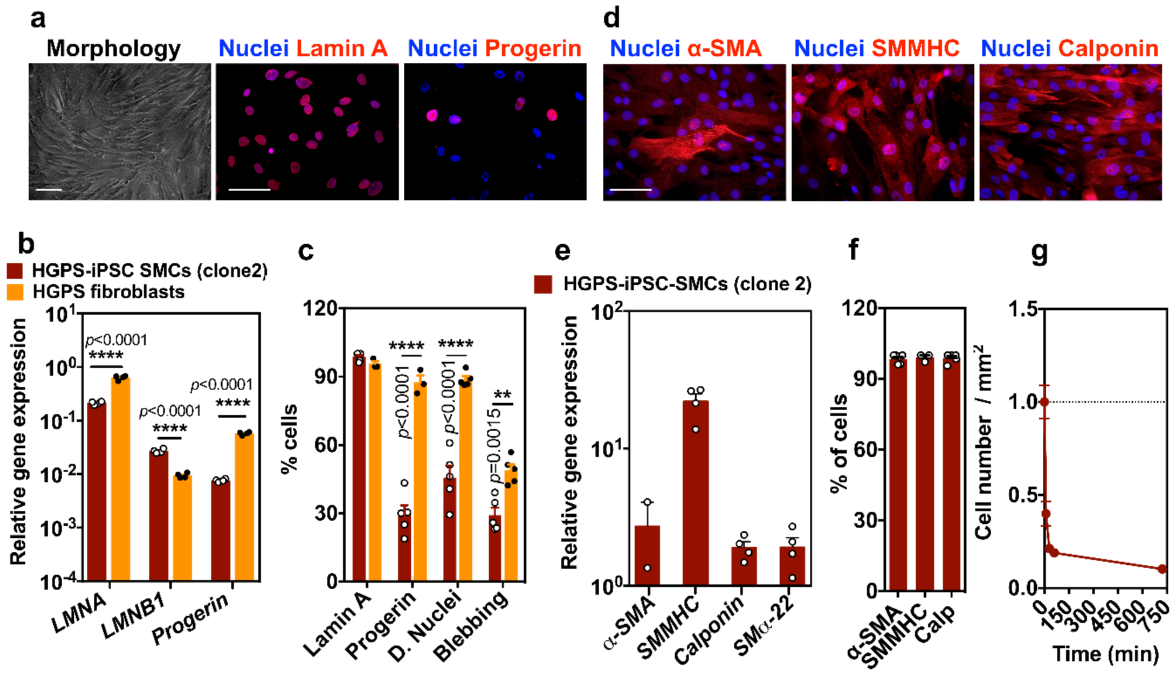

Supplementary Figure 5: Expression of progeria and SMC markers in HGPS-iPSC SMCs (second individual). a Expression of progeria proteins by immunofluorescence. Scale bar is 50 µm. n=3-5 independent experiments. **b** Expression of progeria gene markers. n=4 technical replicates from a pool of 3 independent experiments. **c** Expression of progeria markers (proteins and morphology). n=3-5 independent experiments. In **b** and **c**, results are Mean ± SEM. *,**, ***, **** denotes statistical significance ($p<0.05$, $p<0.01$, $p<0.001$ and $p<0.0001$). Statistical analyses were performed by two-tailed unpaired Student's t-test. **d** Expression of SMC proteins by immunofluorescence. Scale bar is 50 µm. n=5 independent experiments except for *SMMHC* (n=4). **e** Expression of SMC gene markers. n=4 technical replicates from a pool of 3 independent experiments. **f** Percentage of cells that express SMC markers at protein level. n=5 independent experiments except for *SMMHC* (n=4). In **e** e **f**, results are Mean ± SEM. **g** Number of cells per surface (mm^2) during cell culture under flow (at least 3 images (x10) have been quantified per time). Time under flow shear stress in minutes. Results are Mean ± SEM (n=3 independent experiments except for time 0 and 10 min (n=4)).

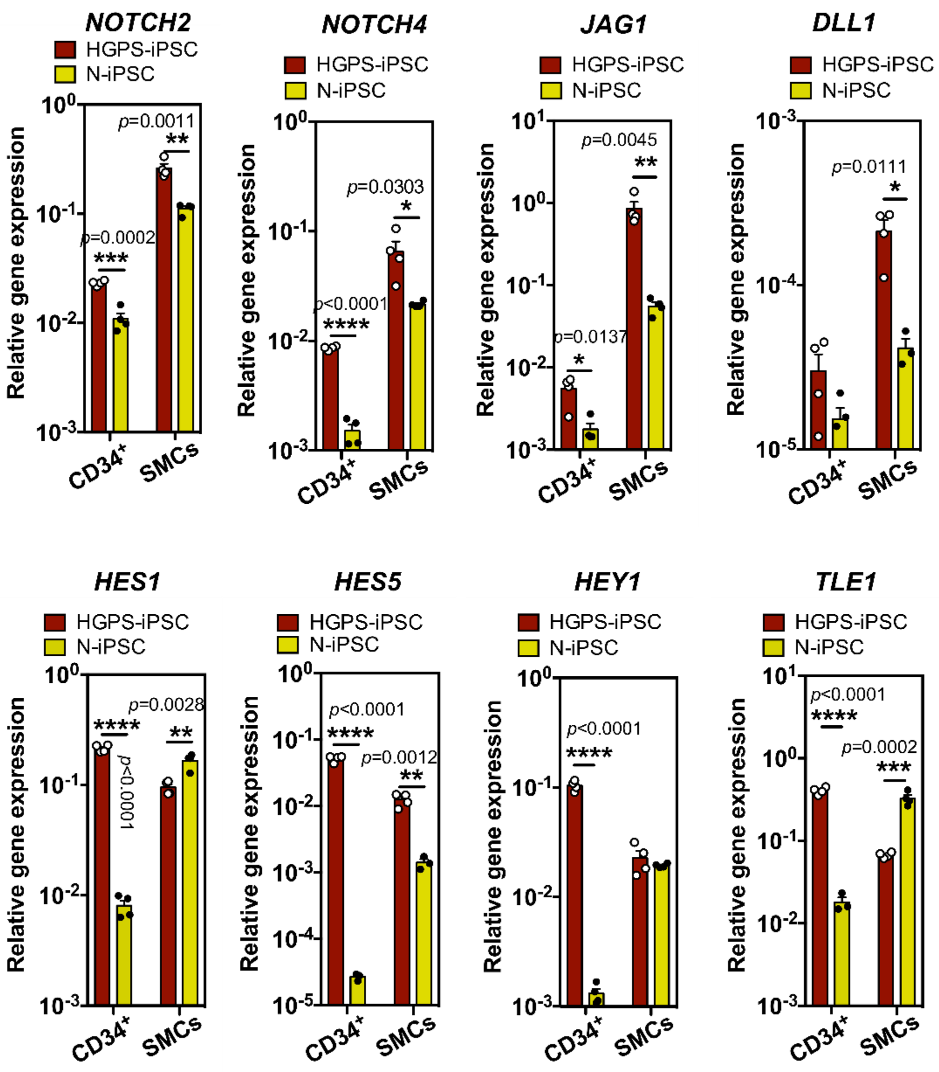

Supplementary Figure 6: Activation of signaling pathways during the differentiation of HGPS-iPSCs into SMCs. Expression of NOTCH upstream (*NOTCH2*, *NOTCH4*, *JAG1* and *DLL1*) and downstream (*HES1*, *HES5*, *HEY1*, *TLE1*) effector genes, as evaluated by qRT-PCR during differentiation of CD34+ cells into SMCs. Values were normalized to the housekeeping gene *GADPH*. Results are Mean ± SEM (n=4 technical replicates from a pool of 3 independent experiments). *,**,***,**** denotes statistical significance ($p<0.05$, $p<0.01$, $p<0.001$, $p<0.0001$, respectively). Statistical analyses were performed by two-tailed unpaired Student's t-test. Our results indicate that HGPS-iPSC CD34+ cells have a higher expression of up- and downstream NOTCH signaling pathway genes than N-iPSC CD34+ cells. In addition, with the exception of downstream signaling NOTCH genes such as *HES1*, *HEY1* and *TLE1*, HGPS-iPSC SMCs have a higher expression of *NOTCH* genes than N-iPSC SMCs.

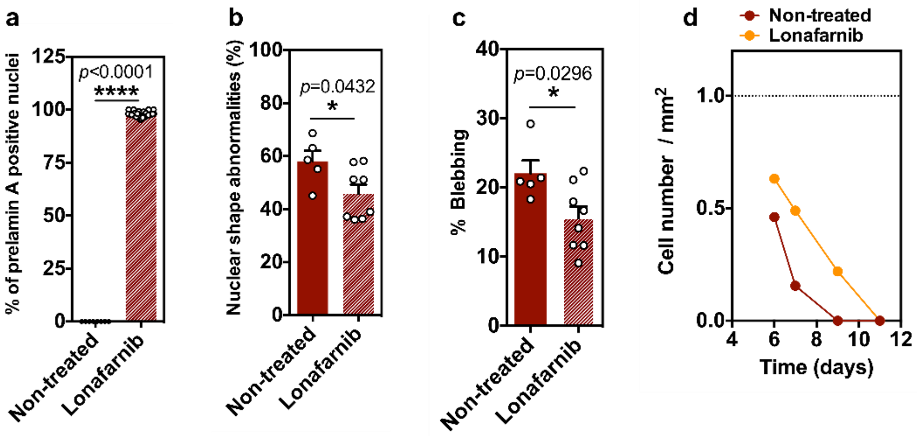

Supplementary Figure 7: Response of HGPS- iPSC SMCs to a farnesyltransferase inhibitor, lonafarnib. a Accumulation of prelamin A in HGPS-iPSC SMCs treated with lonafarnib (20 μM). n=9 images examined over 4 independent experiments. Nuclear abnormalities **b** and nuclear blebbing (n=3 images examined over 2 or 3 independent experiments). **c** in HGPS-iPSC SMCs cultured with lonafarnib (n=3 images examined over 2 or 3 independent experiments). **d** Effect of lonafarnib in cell number cultured under flow conditions (n=4 independent experiments). In a-d results are Mean ± SEM. *,**,*** denotes statistical significance (p<0.05, p<0.01, p<0.001). Statistical analyses were performed by two-tailed unpaired Student's t-test.

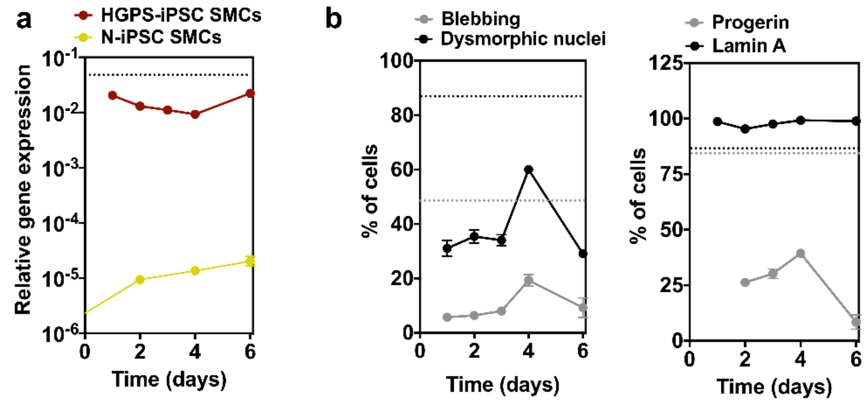

Supplementary Figure 8: Characterization of HGPS-SMCs under flow culture conditions. a Progerin expression in N-iPSC SMCs and HGPS-iPSC SMCs. Gene expression was normalized by the housekeeping gene *GAPDH*. Gene expression in HGPS Fibroblasts is represented as a dashed line. n=4 technical replicates from a pool of 3 independent experiments. **b** Cellular expression of progeria markers (blebbing, dysmorphic nuclei, progerin and lamin A) as evaluated by immunofluorescence (at least 100 cells were counted). The expression of progeria markers in HGPS Fibroblasts is represented in the graph by a dashed line. n=3 independent experiments. In **a** and **b**, results are Mean ± SEM.

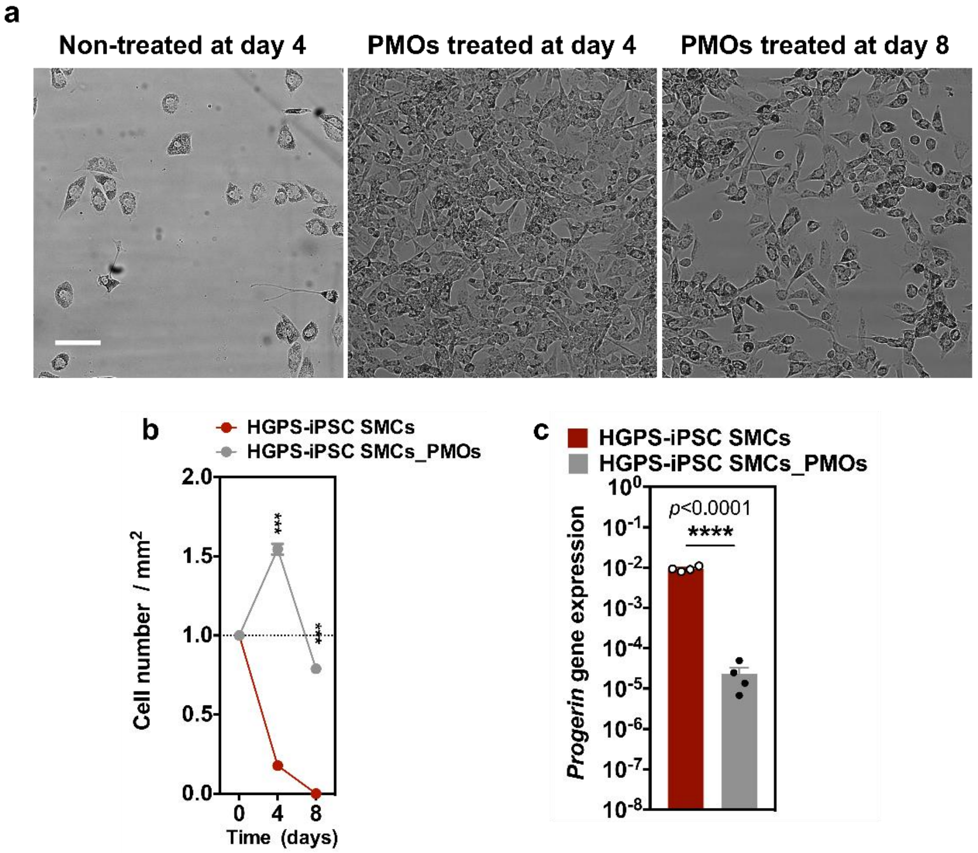

Supplementary Figure 9: Effect of progerin inhibition on SMC vulnerability to flow shear stress. HGPS-iPSC SMCs were seeded overnight in the microfluidic system and then perfused with SmGM-2 medium supplemented with PMOs (Ex10 and Ex11 at 20 µM each) at arterial flow rate (20 dyne/cm^2). After 48 h the medium was replaced by new medium supplemented with PMOs. Nontreated cells were used as negative control. **a** Cell morphology and number observed by light microscopy. Scale bar is 50 µm and n=6 independent experiments. **b** Number of cells per surface area (mm^2) (at least 6 images (x20) have been quantified per condition). Results are Mean ± SEM (n=6 independent experiments). **c** *Progerin* gene expression at day 4 evaluated by qRT-PCR. Gene expression was normalized by the housekeeping gene *GAPDH*. Results are Mean ± SEM (n=4 technical replicates from a pool of 3 independent experiments). For **b** and **c**, **** denotes statistical significance (p<0.0001). Statistical analyses were performed by two-tailed unpaired Student's t-test.

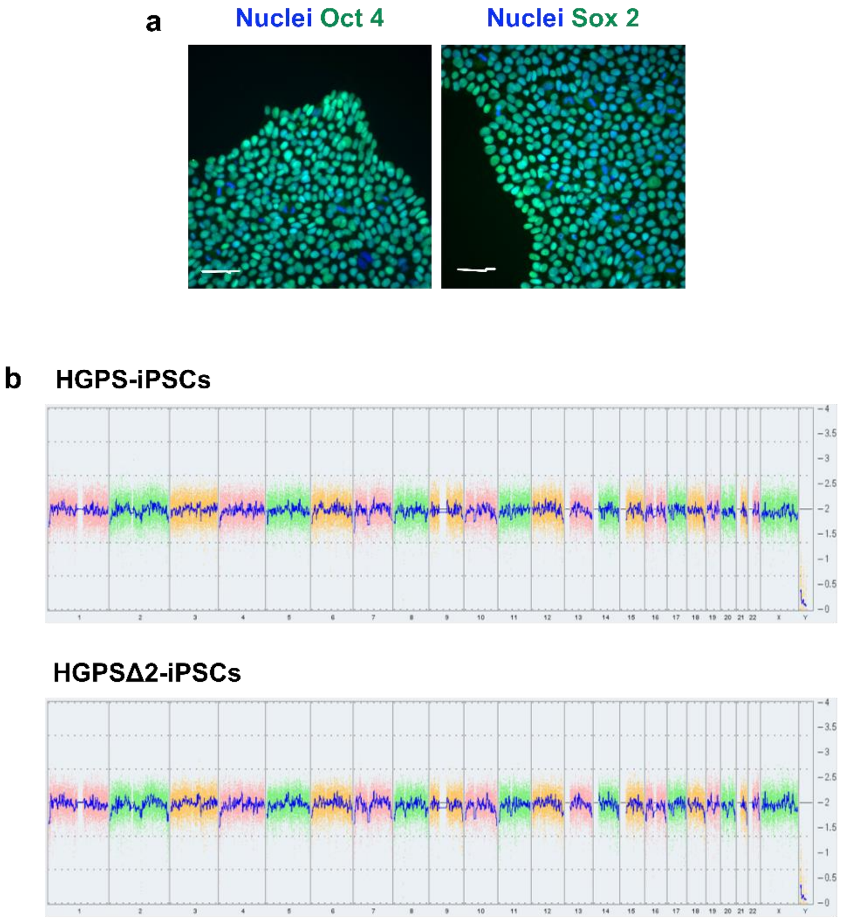

Supplementary Figure 10: Characterization of gene edited HGPS-iPSCs. a Pluripotency marker expression. Scale bars indicate 50 µm. n=3 independent experiments. **b** Karyotype profile in HGPSΔ2-iPSCs, analyzed using KaryoStatTM assay.

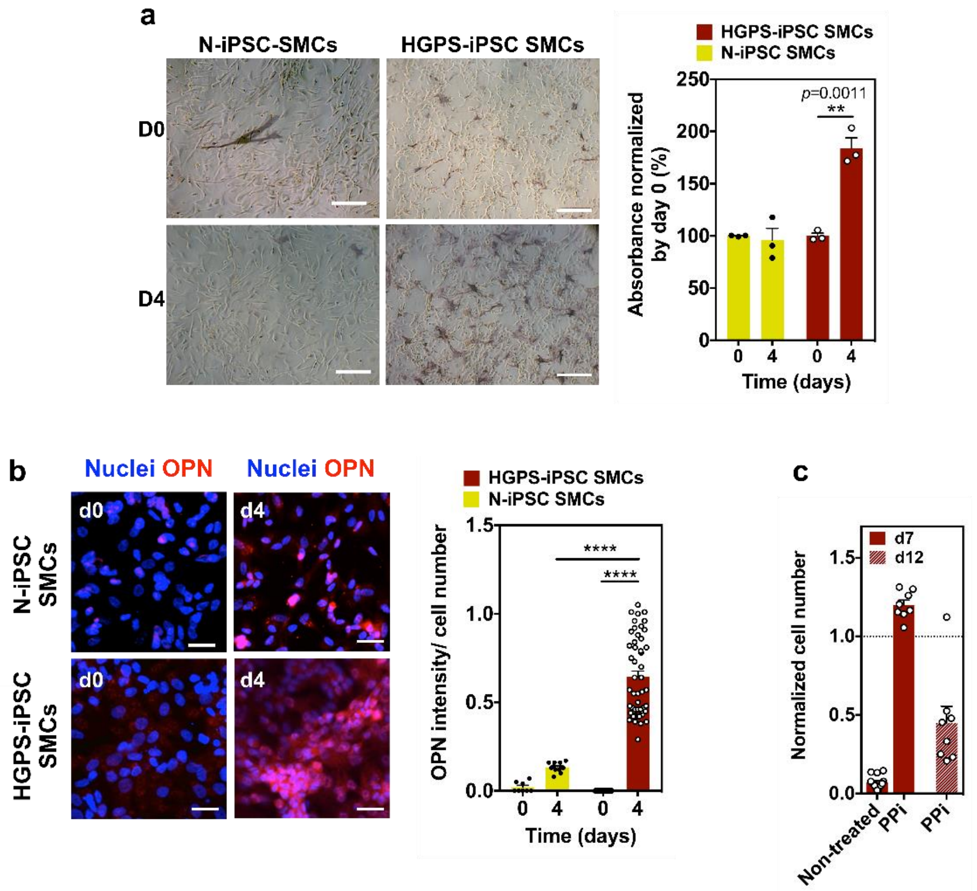

Supplementary Figure 11: HGPS-iPSC SMCs cultured under arterial flow conditions show an osteogenic differentiation program. Because arteries in HGPS individuals have high probability to be calcified, we evaluated whether HGPS-iPSC SMCs could undergo an osteogenic differentiation program after culture in flow conditions. **a** Alkaline phosphatase activity in HGPS-iPSC SMCs and N-iPSC-SMCs cultured in flow conditions (brownish areas are linked to activity of alkaline phosphatase) in SmGM2 media (non-osteogenic) for 4 days. Scale bar is 200 µm. n=3 independent experiments. Quantification of alkaline phosphatase activity by a colorimetric assay and normalized by cell number per mm^2 at day 4. n=3 independent experiments. Statistical analyses were performed by two-tailed unpaired Student's t-test. **b** Expression of osteopontin (OPN) in HGPS-iPSC SMCs cultured in flow conditions in SmGM2 media (non-osteogenic) at day 4. Scale bar is 50 µm. n=4 independent experiments. The intensity of OPN was measured and data normalized by cell number. n=2-13 images examined over 4 independent experiments. Statistical analyses were performed by one-way ANOVA followed by Newman Keuls's post-test. **c** Effect of calcification inhibitor (PPi) in HGPS-iPSC SMCs detachment. The number of cells was evaluated after 7 and 12 days under arterial flow and was normalized by the surface area (mm^2). n=2-3 images examined over 4 independent experiments. In **a, b** and **c**, results are Mean ± SEM. *,**,*** denotes statistical significance ($p<0.05$, $p<0.01$, $p<0.001$). Our results indicate that HGPS-iPSC SMCs had higher alkaline phosphatase activity and osteopontin expression than N-iPSC-SMCs.

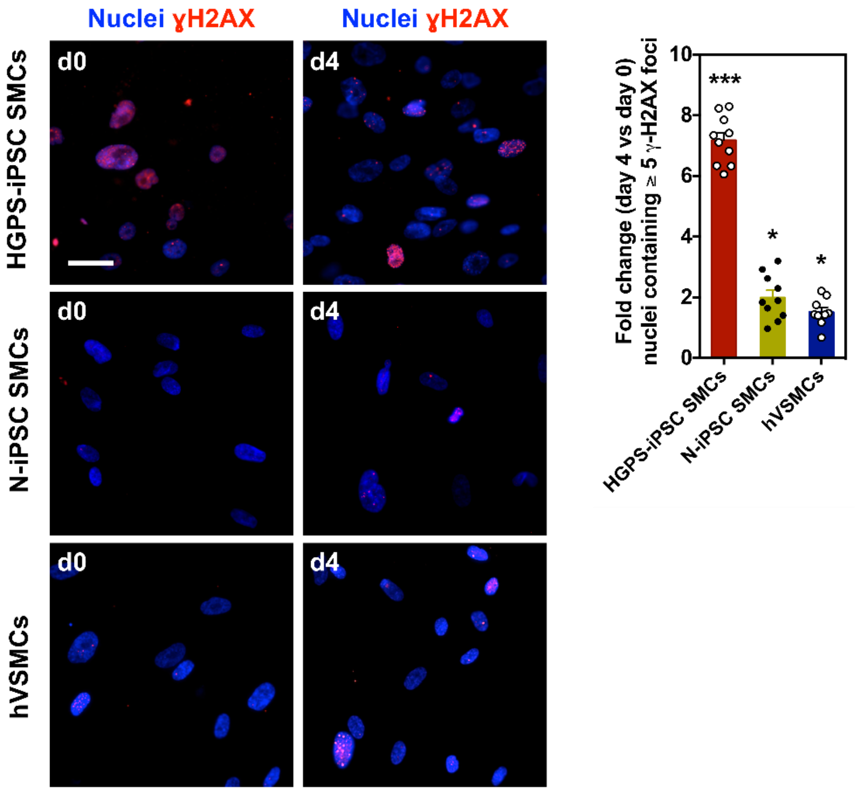

Supplementary Figure 12: Expression of ɣH2AX, a marker for DNA damage. Immunofluorescence analyses for ɣH2AX in HGPS-iPSC SMCs, N-iPSC SMCs and hVSMCs. n=4 independent experiments. Scale bar is 50 µm. For quantification, 3 slides have used per condition. In each slide, 2-3 images were analyzed and more than 100 cells counted for the presence of 5 or more ɣH2AX foci. The fold change between cells cultured under flow conditions during 4 days and cells cultured in static conditions (day 0) was calculated. Results are Mean ± SEM (n=2-3 images examined over 4 independent experiments). *,*** denotes statistical significance (p<0.05, p<0.001) relatively to day 0.

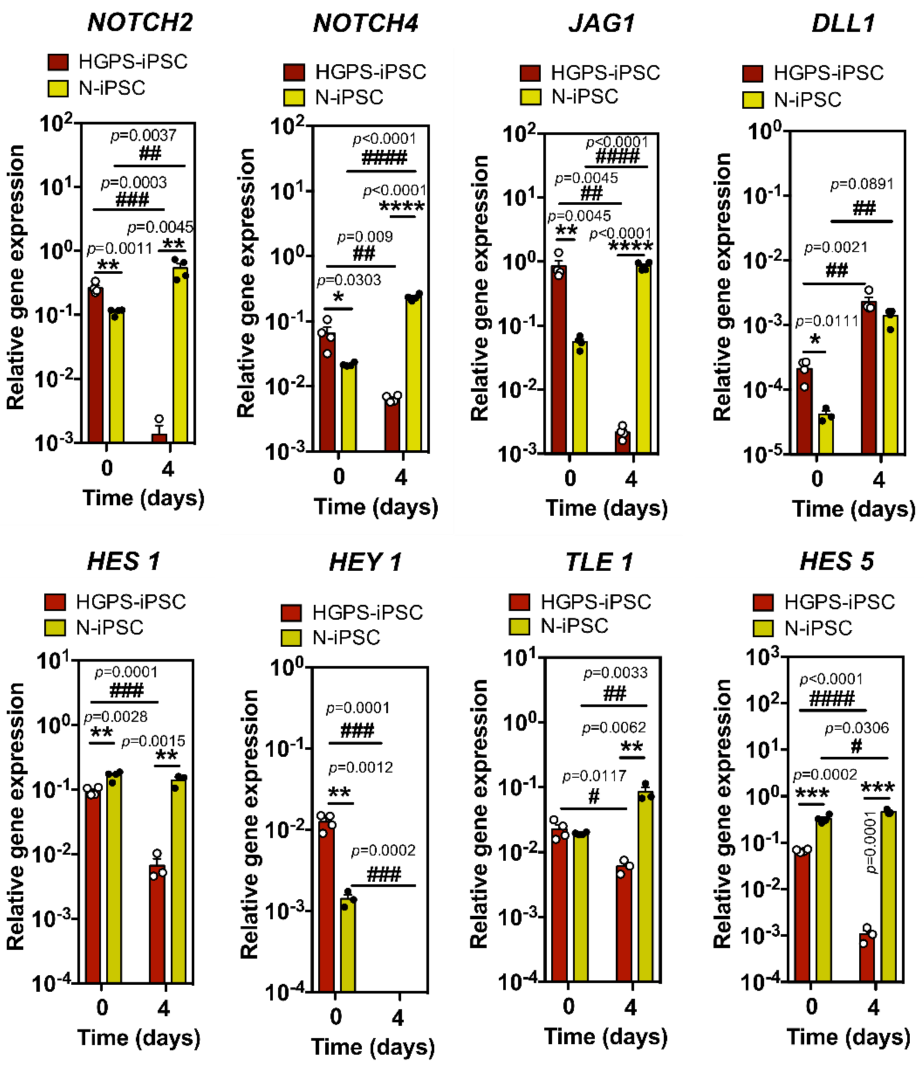

Supplementary Figure 13: Expression of NOTCH signaling pathway on HGPS-iPSC-SMCs cultured under flow conditions. Expression of NOTCH signaling effectors as evaluated by qRT-PCR, in HGPS-iPSC-SMC and N-iPSC-SMCs cultured under flow conditions. Results are Mean ± SEM (n=4 technical replicates from a pool of 3 independent experiments). *,**,***,**** denotes statistical significance (p<0.05, p<0.01, p<0.001, p<0.0001) compared between HGPS-iPSC and N-iPSC for each day. #, ##, ###, ### denotes statistical significance (p<0.05, p<0.01, p<0.001, p<0.0001) between HGPS-iPSC d0 and d4, or N-iPSC d0 and d4. Gene expression was normalized by the housekeeping gene *GAPDH*. Statistical analyses were performed by two-tailed unpaired Student's t-test.

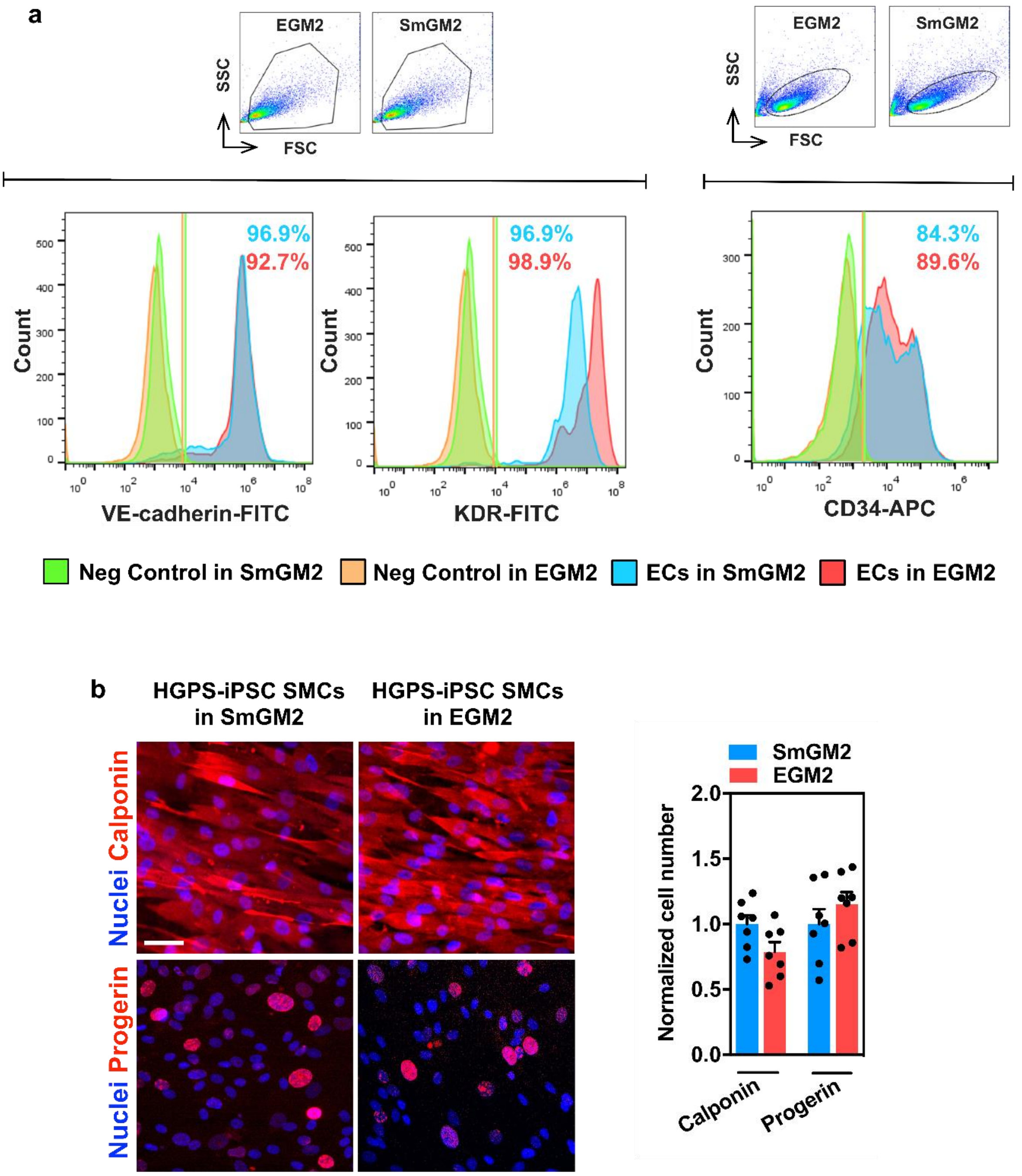

Supplementary Figure 14: Effect of cell culture medium in EC and HGPS-iPSC SMC phenotype. a Flow cytometry analyses of EC markers in HUAECs cultured for 3 days in EGM2 or SmGM2 media. Cells were initially gated in an FSC/SSC plot (attached to the histogram plot) and then gated for the expression of EC markers. **b** Immunofluorescence analyses for calponin and progerin in HGPS-iPSC SMCs cultured for 3 days in EGM2 or SmGM2 media. Scale bar is 50 µm. n=3 independent experiments. Number of HGPS-iPSC SMCs expressing calponin and progerin. Results are Mean ± SEM (n=2-3 images examined over 3 independent experiments).

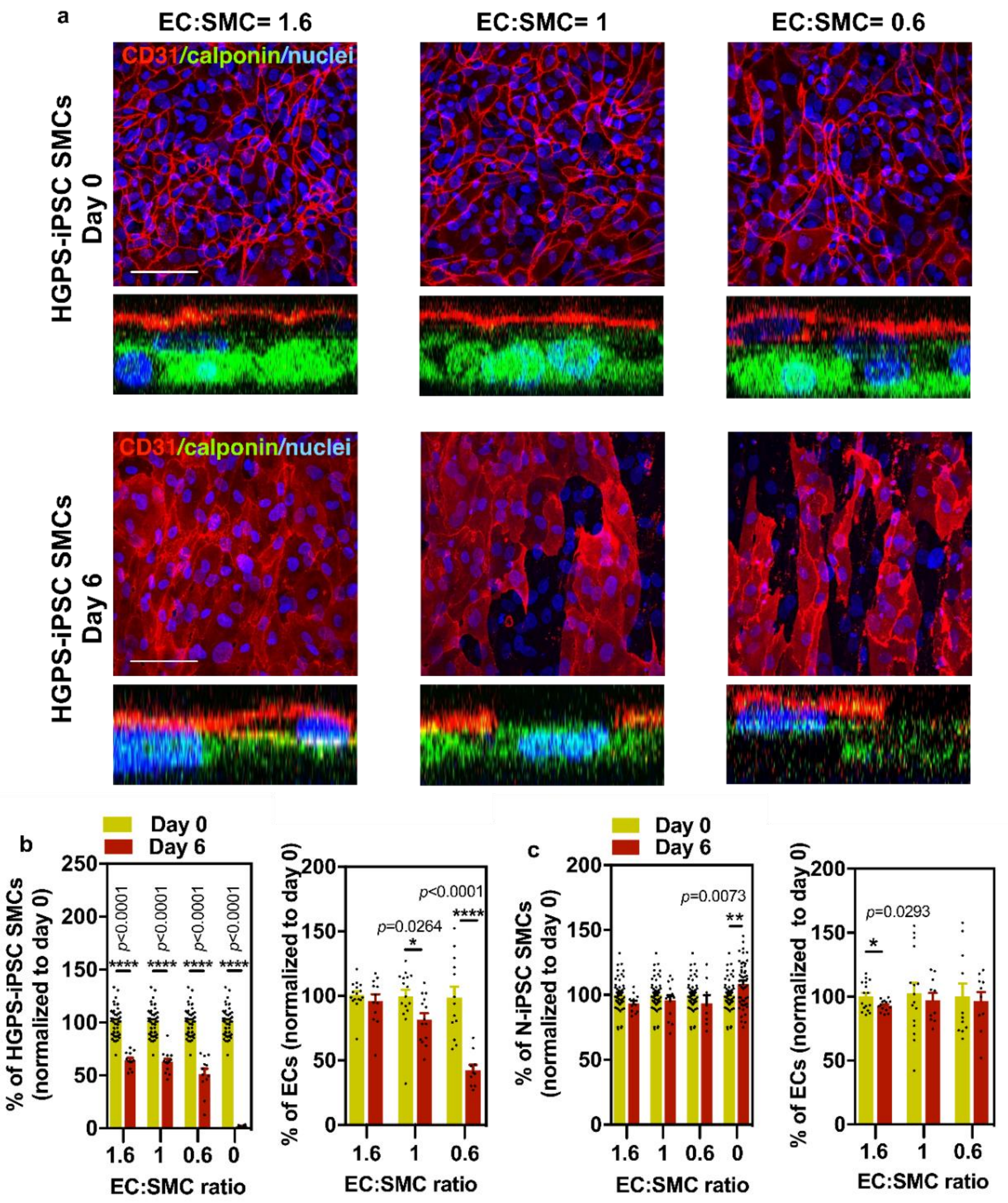

Supplementary Figure 15: Effect of flow in a co-culture of HGPS-iPSC SMCs with ECs. HGPS-iPSC SMCs and N-iPSC SMCs were seeded in a microfluidic channel for 4 h followed by the seeding of ECs on top of the SMCs for 4 h after which the cells were cultured under flow conditions (20 dyne/cm^2). **a** Immunofluorescence analyses. ECs, SMCs and cell nuclei were stained with CD31, calponin and DAPI, respectively. Scale bar is 100 µm. n=3 independent experiments. Orthogonal view of a z-stack confocal images showing the presence of 2 layers of cells at day 0 and dysfunctional layers after 6 days under flow conditions. **b** Quantification of HGPS-iPSC SMCs and ECs at days 0 and 6. (n=3-16 images examined over 3 independent experiments) **c** Quantification of N-iPSC SMCs and ECs at days 0 and 6 (n=3-16 images examined over 3 independent experiments). In b and c, results are Mean ± SEM. * and **** denotes statistical significance ($p<0.05$ and $p<0.001$). Statistical analyses were performed by two-tailed unpaired Student's t-test.

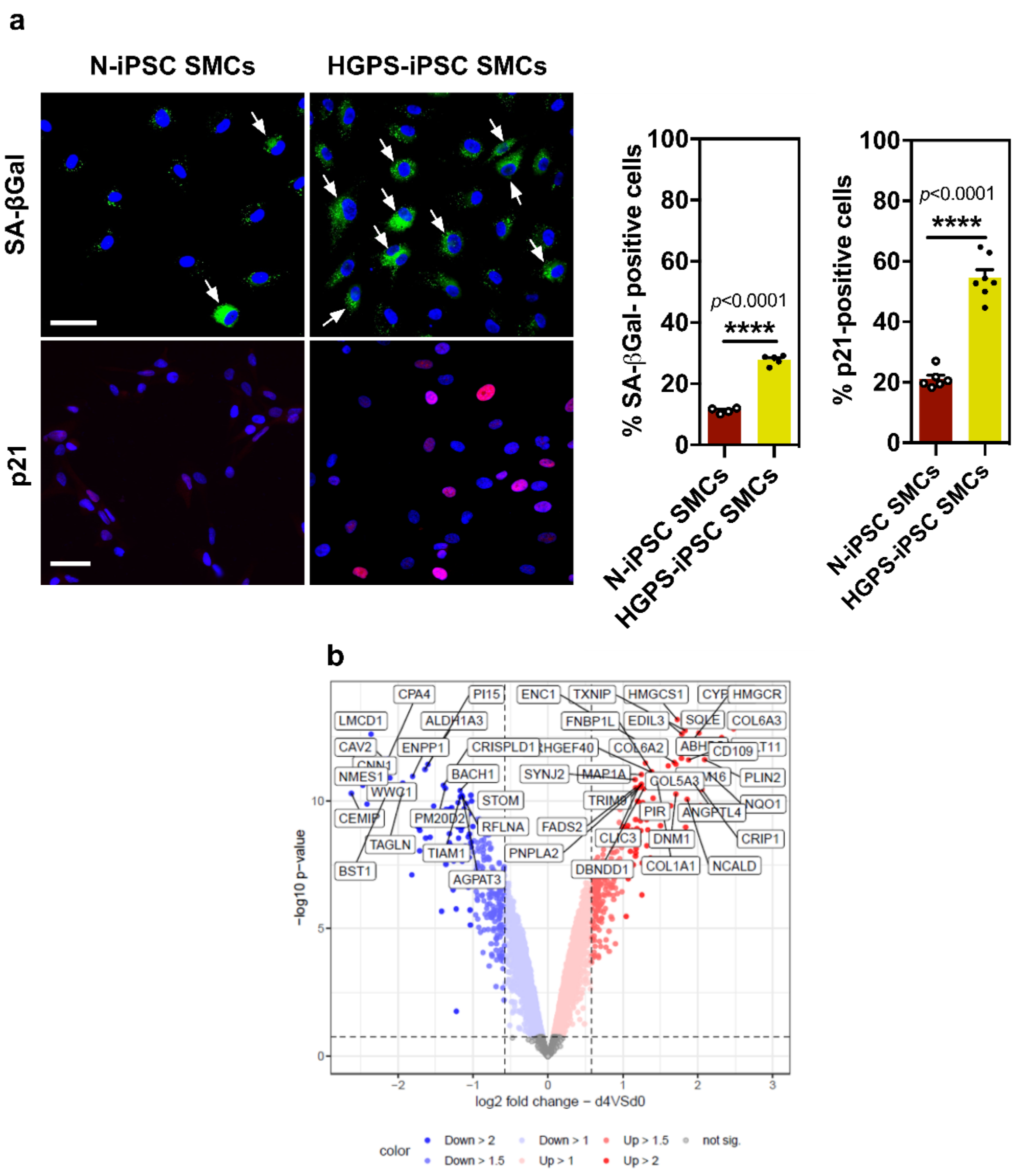

Supplementary Figure 16: Senescence phenotype of HGPS-iPSC SMCs. a Expression of SA-βGal and p21 in HGPS-iPSC SMCs by immunofluorescence. Representative images and quantification. Scale bar is 50 µm. Results are Mean ± SEM (n=1-3 images examined over 3 independent experiments). **** denotes statistical significance (p<0.0001). Statistical analyses were performed by two-tailed unpaired Student's t-test. **b** Volcano plot representing differentially expressed proteins in HGPS-iPSC-SMCs compared cells cultured under flow condition during 4 days and cells cultured under static condition (day 0). Each point represents one of 6997 proteins.

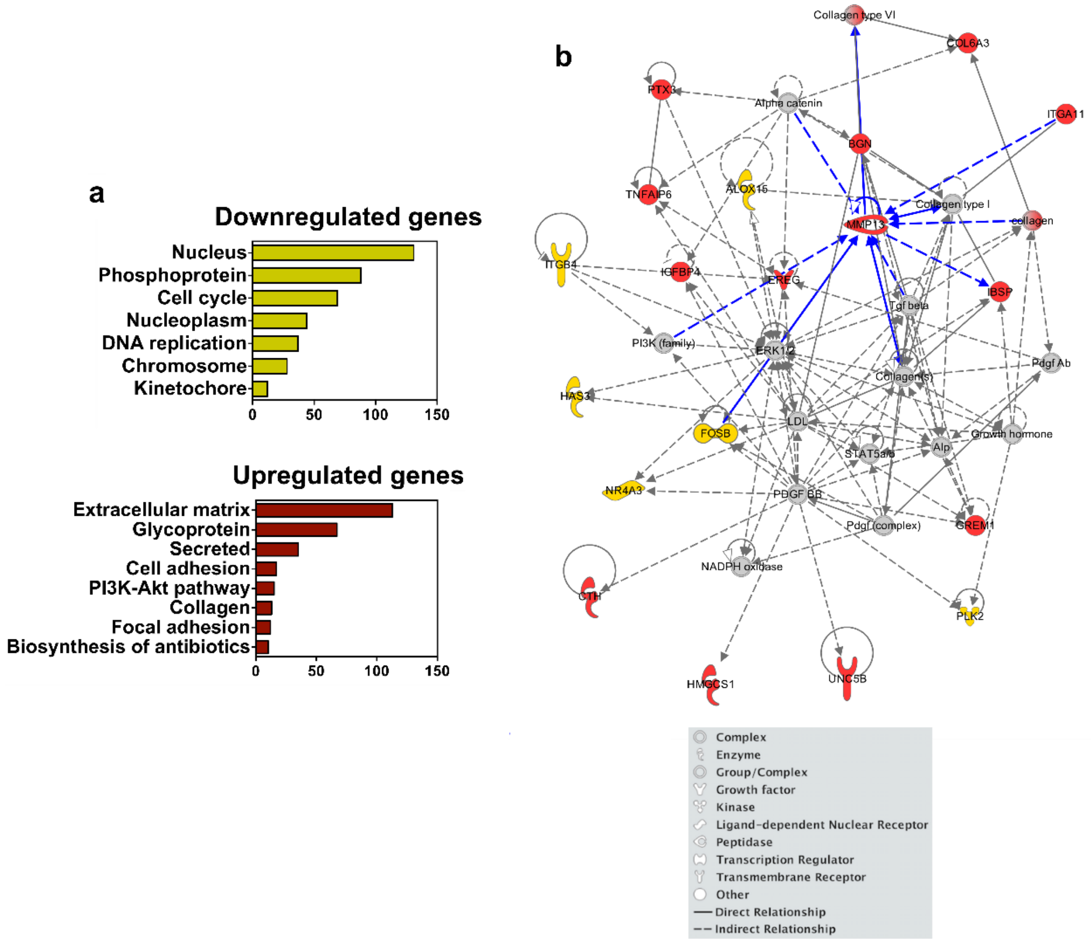

Supplementary Figure 17: Gene expression profile in HGPS-iPSC SMCs cultured under flow shear stress. a DAVID functional enrichment analysis; data obtained from down and upregulated genes for the HGPS-iPSC SMC cells under arterial stress conditions at day 4. Only genes with a Log fold change > 1 and p-value < 0.05 were chosen. **b** Ingenuity Pathway Analysis; differentially expressed genes under arterial stress conditions at day 4 in HGPS-iPSC SMC cells. Genes with a fold change >= 3 and p-value 0.05 were selected.

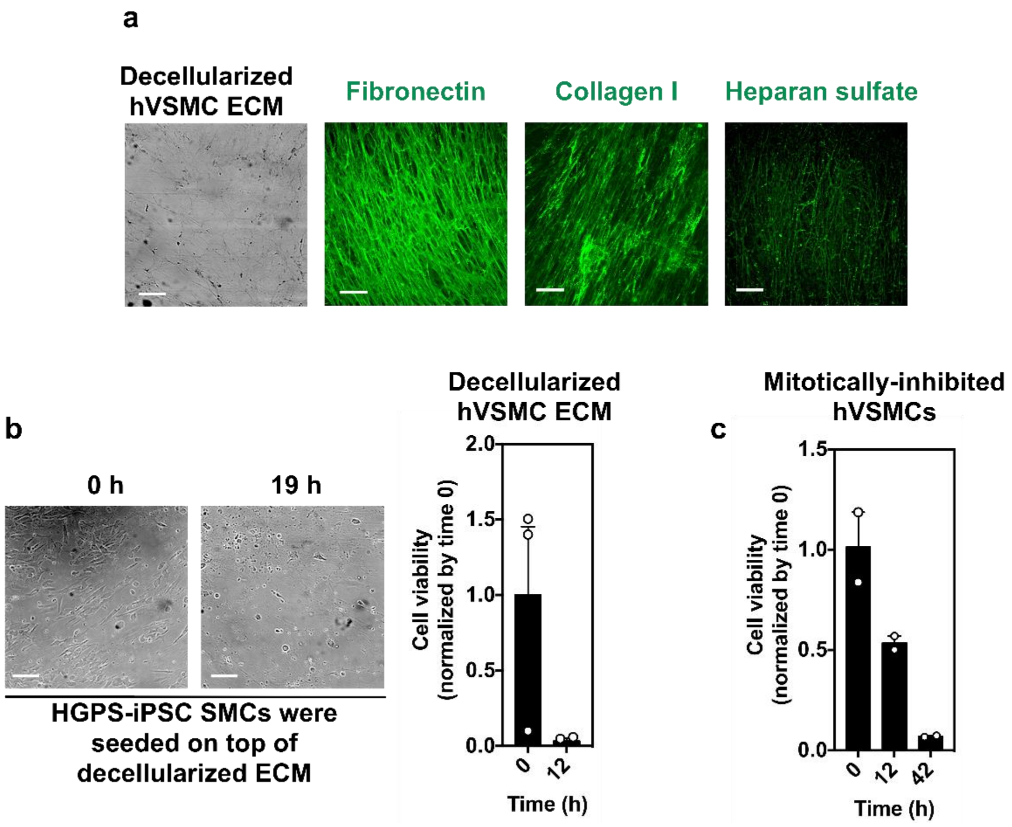

Supplementary Figure 18: Effect of ECM on SMC vulnerability to flow shear stress. a Decellularized ECM from hVSMCs was used as substrate for the culture of HGPS-iPSC SMCs in flow conditions. The decellularized ECM was composed by fibronectin, collagen type I and heparan sulfate. Unfortunately, the substrate was unable to prevent the detachment of HGPS-iPSC SMCs after 12-19 h in flow conditions. Scale bar is 50 µm and n=3 independent experiments. **b** Cell viability was evaluated by Presto Blue. Results are Mean ± SEM (n=3 independent experiments). Scale bar is 50 µm. **c** HGPS-iPSC SMCs were seeded on mitotically-inhibited hVSMCs and exposed to flow conditions. Cell viability was evaluated by Presto Blue. After 2 days, most of the cells detached. Results are Mean ± SEM (n=2 independent experiments).

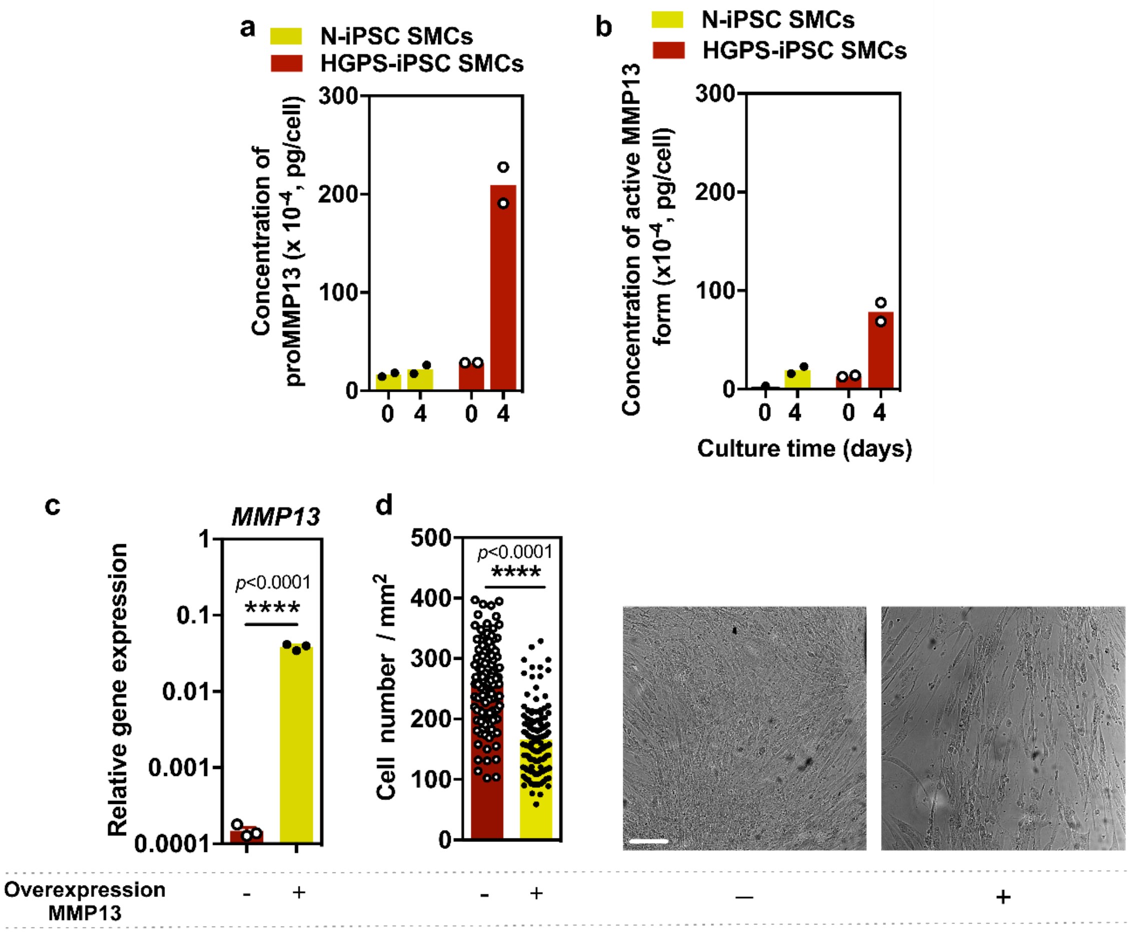

Supplementary Figure 19: Overexpression of MMP13 in hVSMCs. Quantification of total Pro-MMP13 (**a**) and endogenous active form of MMP13 (**b**) were accessed by ELISA. Cells were analyzed at day 0 and day 4 under flow. Fluorescence signal was normalized by cell number. In a and b, results are Mean ± SEM (n=2 independent experiments). * denotes statistical significance (p<0.05). **c** MMP13 lentiviral particles were used to induce the expression of MMP13 in hVSMC. The expression of *MMP13* was analyzed by qRT-PCR at day 3 post-infection. Results are Mean ± SEM (n=3 independent experiments). **** denotes statistical significance (p<0.0001). Statistical analyses were performed by two-tailed unpaired Student's t-test. Gene expression was normalized by the housekeeping gene *GAPDH*. **d** hVSMCs overexpressing MMP13 were cultured under flow conditions for 7 days. After cell attachment and before flow conditions, the number of cells per area of substrate was around 260 cell/mm^2. At the end, the number of cells per area of substrate was quantified by high content microscopy (cell nuclei were stained with Hoechst dye). Scale bar is 50 µm. Results are Mean ± SEM (n=37 images examined over 3 independent experiments). **** denotes statistical significance (p<0.0001). Statistical analyses were performed by two-tailed unpaired Student's t-test.

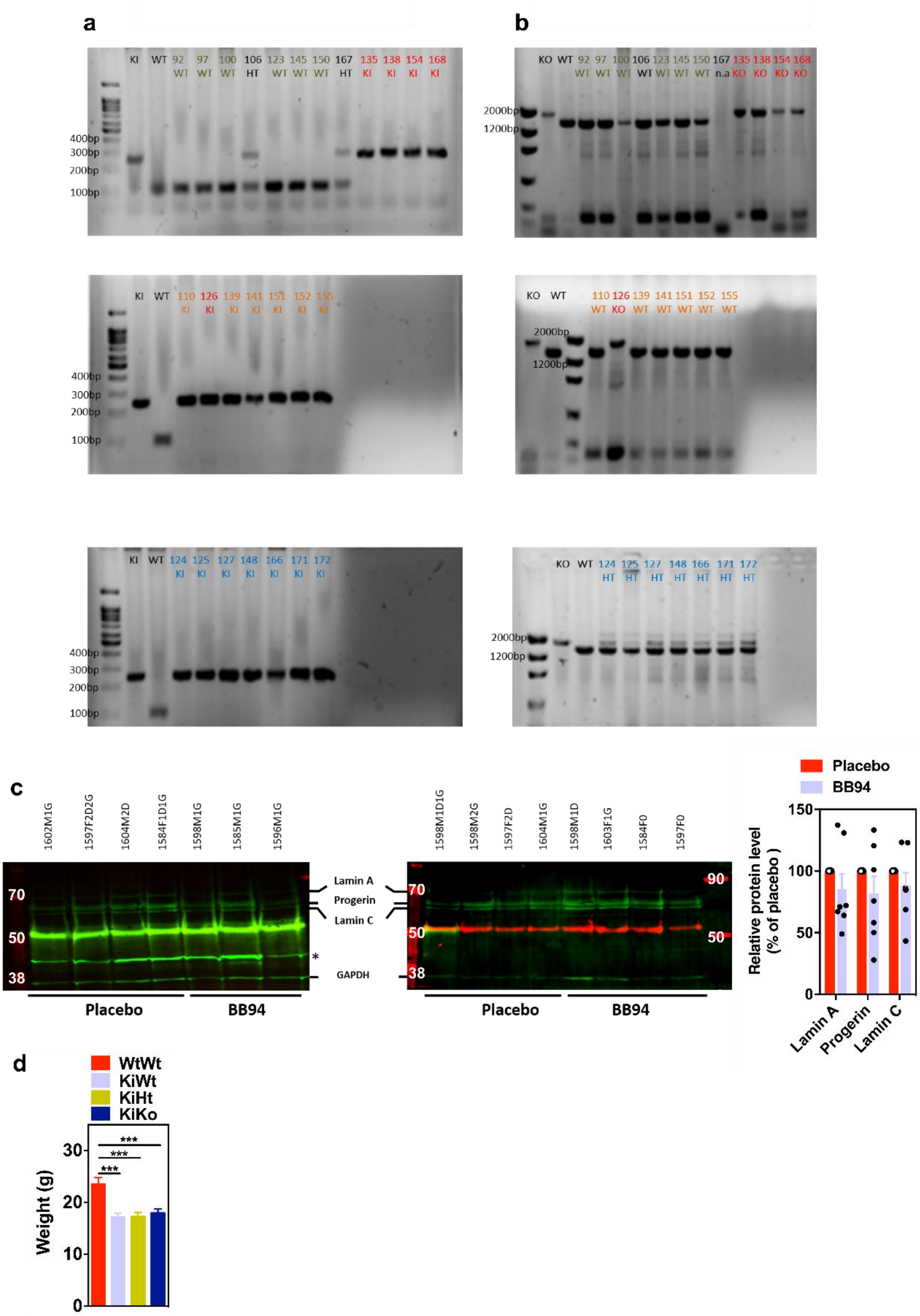

Supplementary Figure 20: Progerin and MMP13 expression in mice. a, b Genomic DNA from tails tips was used to perform PCR for the detection of *Lmna* (**a**) and *Mmp13* (**b**) expression. The *Lmna* approximate fragment sizes of each band were 100 bp for wild type (WT) *Lmna* allele, and 240 bp for excised G609G allele (knock in (KI)) on agarose gels. The *Mmp13* approximate fragment sizes of each band were 1300 bp for wild type (WT) *Mmp13*,

and 1485 bp for *Mmp13* knockout (KO) mice on agarose gels. HT were heterozygous mice for *Lmna* and/or *Mmp13*. For **a**, n=6 Wt, n=2 Ht, n=18 Ki animals. For **b**, n=13 Wt, n=7 Ht, n=5 Ko animals. **c** Quantification of progerin in mice treated or not with BB94. Progerin levels were evaluated by western blot. n=7 animals. Results are Mean ± SEM (n=7 animals) **d** Body weight in mice (n=5 WtWt mice, n=6 KiWt mice, n=7 KiHt mice and n=5 KiKo mice). Statistical analyses were performed by one-way ANOVA followed by Newman Keuls's post-test.

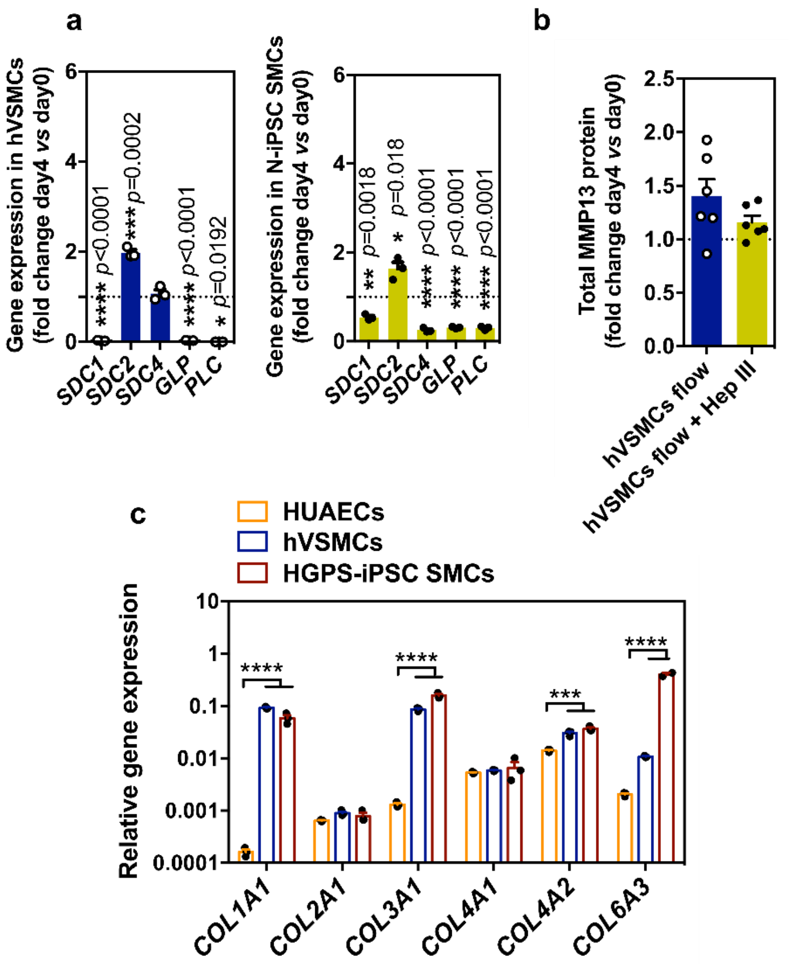

Supplementary Figure 21: Expression of glycocalyx and ECM components in normal and HGPS-iPSC SMCs. **a** Cells (N-iPSC SMCs and hVSMCs) were cultured under flow conditions for 4 days and gene expression of glycocalyx markers (*SDC1*: syndecan 1, *SDC2*: syndecan 2, *SDC4*: syndecan 4, *GPC*: glypican, *PLC*: perlecan) were evaluated by qRT-PCR. Gene expression was normalized by the housekeeping gene *GAPDH*, and the normalized gene expression at day 4 divided by day 0. Results are Mean ± SEM (n=3 technical replicates from a pool of 3 independent experiments) Statistical analyses were performed by two-tailed unpaired Student's t-test. relatively to day 0. **b** hVSMCs cultured under flow condition were treated or not with heparinase III and MMP13 protein was calculated by ELISA and normalized by the number of cells present at day 0. n=2 technical replicates over 3 independent experiments. **c** Quantification of collagen mRNA transcripts in cells cultured in static conditions for 4 days. Gene expression was normalized by the housekeeping gene *GAPDH*. Results are Mean ± SEM (n=3 technical replicates from a pool of 3 independent experiments). ***,**** denotes statistical significance (p<0.001 and p<0.0001). Statistical analyses were performed by one-way ANOVA followed by Newman Keuls's post-test.

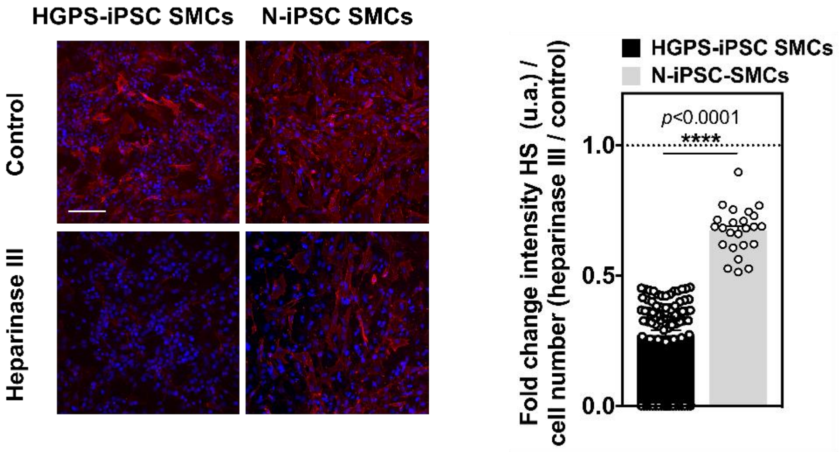

Supplementary Figure 22: Effect of heparinase III on heparan sulfate. Cells were seeded overnight in the microfluidic system and then treated with heparinase III 0.5 U/mL for 30 min. After the treatment cells were perfused with SmGM-2 at arterial flow rate (20 dyne/cm^2). After 2 days, heparan sulphate intensity was measured using ImageJ and normalized by cell number. The fold change between cells treated with heparinase III and control cells was calculated. For quantification, 3 slides have used per condition. Results are Mean ± SEM (n=8 or 27 images examined over 3 independent experiments). Scale bar in confocal microscope images is 50 µm. **** denotes statistical significance (p<0.0001). Statistical analyses were performed by two-tailed unpaired Student’s t-test.

Supplementary Table 1. Specific sets of primers and Probe (FAM) for Progerin using Taqman technology. Taqman PCR conditions: initial step at 50 ºC for 2 min; after another step at 95 ºC 10 min; 45 cycles at 95 °C for 15 sec. and at 60 ºC for 1 min. After amplification, melting curves were acquired and used to determine the specificity of PCR products.

	**Probe**	**Sense**	**Antisense**
Progerin	CGGGAGCCCAGAGCTCCCAGAA	TGAGTACAACCTGCGCTCAC	TGGCAGGTCCCAGATTACAT

Supplementary Table 2. Specific sets of primers designed by Sigma. SYBR Green PCR conditions: initial denaturation step at 94 ºC for 5 min; 40 cycles of denaturation at 94 °C for 30 sec. annealing at 60ºC for 33 sec and extension at 72 ºC for 30 sec. At the end was performed a final 7 minutes extension at 72 ºC. After amplification, melting curves were acquired and used to determine the specificity of PCR products.

	**Sense**	**Antisense**
AMTN	TAATCCAGATGTCCAGGATG	TTCCATTTGCTGATTCTGTG
ANGPTL4	AGGCAGAGTGGACTATTTG	CCTCCATCTGAGGTCATC
BGN	GGTGGTCTATCTGCACTC	TGATGCCGTTGTAGTAGG
CALPONIN	TTTTGAGGCCAACGACCTGT	TCCTTTCGTCTTCGCCATG
COL6A3	GTCATTGAAGTCAACAAGAGAG	GATAAGATCCTGTCCGATTTC
COL1A1	GCTGTCTTATGGCTATGATGAG	AGACCACGAGGACCAGAG
COL2A1	GAAGAGTGGAGACTACTGG	CAGATGTGTTTCTTCTCCTTG
COL3A1	CCTCCTGGAACTGCC	CAACTTCACCCTTAGCAC
COL4A1	AAAGGGAGATCAAGGGATAG	TCACCTTTTTCTCCAGGTAG
COL4A2	AAAAGGAGATAGAGGCTCAC	GTATTCCGAAAAATCCAGCC
DLL1	TCTCTCTTTCTCTTTCTCCC	TCTTTATATCCGCCCTGC
EPPK1	GAGAAGTTGAGGCATACTTG	ATATGACACATAGACGACCC
EPS8	GTAAAGGAGGGAAACAGAAG	ACTCGACTTCTAACATCCAC
GAPDH	AGCCACATCGCTCAGACACC	GTACTCAGCGCCAGCATCG
HAS3	AGATCCTCAACAAGTACGAC	CACTAATACACTGCACACAG
HES1	GCCTATTATGGAGAAAAGACG	CTATCTTTCTTCAGAGCATCC
HES5	AAGAGAAAAACCGACTGC	TTCTCCAGCTTGGAGTTG
HEY1	CCGGATCAATAACAGTTTGTC	CTTTTTCTAGCTTAGCAGATCC
IBSP	GGAGACTTCAAATGAAGGAG	CAGAAAGTGTGGTATTCTCAG
JAG1	GTCTCAAAGAAGCGATCAG	ATATACTCCGCCGATTGG
LIPH	AAATCAATCATGAACCCACC	CAAGGGACCTTAACTTCATTC
LMNA	GGTGGTGACGATCTGGGCT	CCAGTGGAGTTGATGAGAGC
LMNB1	AAGGCGAAGAAGAGAGGTTGAAG	GCGGAATGAGAGATGCTAACACT
MMP13	AGGCTACAACTTGTTTCTTG	AGGTGTAGATAGGAAACATGAG
NOTCH2	AACATCTCATCCATGCTTTG	ACAGTGGTACAGGTACTTC
NOTCH4	ATTGACACCCAGCTTCTTG	GAGGACAAGGGTCTTCAA
NR4A3	AGTTGTCCGTACAGATAGTC	GGTGTTGAGTCTGTTAAAGC
OLFML2A	ATGAACACACTGGAAGAGAC	CAGAGTGATTCTCATAGTGC
PXK	TGAAGGATCTGATCTACAAGG	CCTGAATCTTCTTAGGGTTG
SESN2	CTGACTACTTTACCAGCTTC	TACCAGGTAAGAACACTGATG
SGCG	GTCCCAAAATGGTAGAAGTC	CCCAGTTACTCGAAGTTTATC
SMMHC	CAGGAGTTCCGCCAACGCTA	TCCCGTCCATGAAGCCTTTGG
SMα22	TCCCCGTCCATGAAGCCTTTGG	CGAAAGCCGGCCTTACAGA
TLE1	TATTCCAGTCCAAAGAGTCC	AGATGACTTCATAGACTGTAGC
TNFAIP6	CCAAATGAGTACGAAGATAACC	CACAGTATCTTCCCACAAAG
α-SMA	CCAGCTATGTGAAGAAGAAGAGG	GTGATCTCCTTCTGCATTCGGT
SDC1	TACTAATTTGCCCCCTGAAG	GATATCTTGCAAAGCACCTG
SDC2	AAAGTCACCTGAAGAAACTG	AGACTGTCTGAGTGTTTCTC
SDC4	CAACATCTTTGAGAGAACGG	GCTTTCTTGTAGATGGGTTTC
GPC	AGCTTCGATGACCACTTC	CAGCTCTGAGTACAGGTC
PRLC	TCCTTGAGCTCGTCCCACAAC	GCRGGTGATGCCAAAGCAG

Supplementary Table 3. Specific sets of mouse primers designed by Sigma.

	**Sense**	**Antisense**
Acta2	CATCTTTCATTGGGATGGAG	TTAGCATAGAGATCCTTCCTG
Cnn1	TGCTGAAGTAAAGAACAAGC	CATTGACCTTCTTCACAGAAC
Gapdh	GGAGAAACCTGCCAAGTATGAT	GTGGGAGTTGCTGTTGAAGT
Mmp13	CTTTAGAGGGAGAAAATTCTGG	CATCATCATAACTCCACACG
Myh11	AAAGATGAAAAGTGACCTGC	TTCATTGAAGCCCATGATAG

SUPPLEMENTARY REFERENCES:

1 Vazao, H., das Neves, R. P., Graos, M. & Ferreira, L. Towards the maturation and characterization of smooth muscle cells derived from human embryonic stem cells. *PLoS One* **6**, e17771, doi:10.1371/journal.pone.0017771 (2011).

2 Prewitz, M. C. *et al.* Tightly anchored tissue-mimetic matrices as instructive stem cell microenvironments. *Nat Methods* **10**, 788-794, doi:10.1038/nmeth.2523 (2013).

3 Osorio, F. G. *et al.* Splicing-directed therapy in a new mouse model of human accelerated aging. *Sci Transl Med* **3**, 106ra107, doi:10.1126/scitranslmed.3002847 (2011).

4 Heinze, I. *et al.* Species comparison of liver proteomes reveals links to naked mole-rat longevity and human aging. *BMC Biol* **16**, 82, doi:10.1186/s12915-018-0547-y (2018).

5 Storey, J. D. A direct approach to false discovery rates. *Royal Statistical Society Publications* **64**, 479–498 (2002).

6 Vizcaino, J. A. *et al.* 2016 update of the PRIDE database and its related tools. *Nucleic Acids Res* **44**, 11033, doi:10.1093/nar/gkw880 (2016).

7 McAlister, G. C. *et al.* MultiNotch MS3 enables accurate, sensitive, and multiplexed detection of differential expression across cancer cell line proteomes. *Anal Chem* **86**, 7150-7158, doi:10.1021/ac502040v (2014).

8 Brosch, M., Yu, L., Hubbard, T. & Choudhary, J. Accurate and sensitive peptide identification with Mascot Percolator. *J Proteome Res* **8**, 3176-3181, doi:10.1021/pr800982s (2009).

9 Ritchie, M. E. *et al.* limma powers differential expression analyses for RNA-sequencing and microarray studies. *Nucleic Acids Res* **43**, e47, doi:10.1093/nar/gkv007 (2015).

10 Benjamini, Y. & Hochberg, Y. Controlling the False Discovery Rate: A Practical and Powerful Approach to Multiple Testing. *Journal of the Royal Statistical Society. Series B (Methodological)* **57**, 289-300 (1995).
