# Supplementary material for: Vulnerability of progeroid smooth muscle cells to biomechanical forces is mediated by MMP13
Source: Nat Commun. 2020 Aug 17;11:4110. doi: 10.1038/s41467-020-17901-2 (PMC7431909; doi:10.1038/s41467-020-17901-2)
Supplement: Supplementary file 3 — Description of Additional Supplementary Files [file 41467_2020_17901_MOESM3_ESM.pdf]

Description of Additional Supplementary Files

File Name: Supplementary Data 1
Description: HGPS-iPSC SMC senescence profile at day 0 (before plating the cells in the microfluidic system). 51 Genes were identified from the overlap between the CellAge database (279 genes), and the differentially expressed genes at day 0 on the N-iPSC-SMC control panel versus day 0 on the HPGS-iPSC SMC panel (2084 differentially expressed genes identified; log2FC >= 1, p < 0.05). Statistical analyses were conducted Student's t-test.

File Name: Supplementary Data 2
Description: Reactome Pathway analyses of upregulated differentially expressed genes in HGPS-iPSC SMCs cultured under flow conditions at day 4 versus day 0 (Log2FC >= 1; p < 0.05). Extracellular matrix terms are highlighted.

File Name: Supplementary Data 3
Description: Reactome Pathway analysis of downregulated differentially expressed genes in HGPS-iPSC SMCs cultured under flow conditions at day 4 versus day 0 (Log2FC >= 1; p < 0.05).

File Name: Supplementary Data 4
Description: Differentially expressed proteins in KiWt aortic arch mice compared to WtWt aortic arch mice, and their behavior comparing WtWt vs KiHt and WtWt vs KIKo mice (161 differentially expressed proteins identified; q<0.05 and abs(log2 fold change)>0.58).

File Name: Supplementary Data 5
Description: HGPS-iPSC SMC senescence profile at day 4 (after 4 days under flow conditions) and day 0 (before plating the cells in the microfluidic system) was analyzed by proteomic analyses. 33 proteins were identified from the overlap between the CellAge database (279 genes), and the differentially expressed proteins at day 4 on the HGPS-iPSC-SMC vs day 0 on the HGPS-iPSC SMC panel (1170 differentially expressed proteins identified; log2FC >= 1, p < 0.05). Statistical analyses were conducted Student's t-test.
