# Supplementary material for: Vulnerability of progeroid smooth muscle cells to biomechanical forces is mediated by MMP13
Source: Nat Commun. 2020 Aug 17;11:4110. doi: 10.1038/s41467-020-17901-2 (PMC7431909; doi:10.1038/s41467-020-17901-2)
Supplement: Supplementary file 9 — Reporting Summary [file 41467_2020_17901_MOESM9_ESM.pdf]

natureresearch

Corresponding author(s): Lino Ferreira

Last updated by author(s): May 27, 2020

Reporting Summary

Nature Research wishes to improve the reproducibility of the work that we publish. This form provides structure for consistency and transparency in reporting. For further information on Nature Research policies, see Authors & Referees and the Editorial Policy Checklist.

Statistics

For all statistical analyses, confirm that the following items are present in the figure legend, table legend, main text, or Methods section.

n/a	Confirmed	
☐	☒	The exact sample size (*n*) for each experimental group/condition, given as a discrete number and unit of measurement
☐	☒	A statement on whether measurements were taken from distinct samples or whether the same sample was measured repeatedly
☐	☒	The statistical test(s) used AND whether they are one- or two-sided<br>*Only common tests should be described solely by name; describe more complex techniques in the Methods section.*
☒	☐	A description of all covariates tested
☒	☐	A description of any assumptions or corrections, such as tests of normality and adjustment for multiple comparisons
☐	☒	A full description of the statistical parameters including central tendency (e.g. means) or other basic estimates (e.g. regression coefficient) AND variation (e.g. standard deviation) or associated estimates of uncertainty (e.g. confidence intervals)
☐	☒	For null hypothesis testing, the test statistic (e.g. *F*, *t*, *r*) with confidence intervals, effect sizes, degrees of freedom and *P* value noted<br>*Give P values as exact values whenever suitable.*
☒	☐	For Bayesian analysis, information on the choice of priors and Markov chain Monte Carlo settings
☒	☐	For hierarchical and complex designs, identification of the appropriate level for tests and full reporting of outcomes
☒	☐	Estimates of effect sizes (e.g. Cohen's *d*, Pearson's *r*), indicating how they were calculated

Our web collection on statistics for biologists contains articles on many of the points above.

Software and code

Policy information about availability of computer code

Data collection	- Microscopy images were taken using: Zeiss Labscope v2.8 or InCell Analyser 2200 v6.1 or Zeiss ZEN 2012 SP1 v8.1.0.0.<br>- qRT-PCR was performed using 7500 Fast SDS Software v1.4.1.<br>- Fluidigm data was collected using BioMarkCollection v4.1.1.<br>- Microarray data was collected using GeneAtlas Instrument Control v1.0.5 Software.<br>- For proteomic data acquisition Xcalibur v4.0 (Thermo Scientific) and Tune version 2.9 were employed. For for mass spectrometry, TMT-10plex data were processed using Proteome Discoverer v2.0 (Thermo Fisher). Data were searched against a human database (Uniprot database, Swissprot entry only, release 2016_01) using Mascot v2.5.1 (Matrix Science).<br>- Heart beats were recorded by PhysioSuiteTM non-invasive monitoring system (Kent Scientific Corporation).<br>- For absorbance, fluorescence and luminescence measurements Gen5 v2.05 was used.<br>- The numerical values for widths of medial and adventitial tunicas were obtained using NDP.view2 software (Hamamatsu, Hamamatsu City, Japan).<br>- The flow cytometry analyses were performed in a BD Accuri C6, BD Biosciences.<br>- Karyotype analyses, chips were washed and stained simultaneously using GeneChip Fluidics Station 450 and scanned using GeneChip Scanner 3000 7 G.<br>- Sanger sequencing was performed using the Big Dye Terminator V.1.1 Cycle Sequencing Kit (Applied Biosystems).
Data analysis	- Microscopy images were analyzed using the In Cell Analyser 1000 workstation v3.7.2 or Developer Toolbox v1.9.2 or ImageJ 1.51n.<br>- Prism 6 for windows version 6.01 was used for data analysis.<br>- The raw data from microarrays was analyzed using Expression Console Software from Affymetrix.<br>- Differentially expressed genes from microarrays were identified using the R software at v3.3.1 and the Limma package v3.32 available through Bioconductor (release 3.5).<br>- Reactome Pathway analyses were carried out using Reactome Pathway Analyzer v 61.<br>- Data from Fluidigm was analyzed using Fluidigm Real Time PCR Analysis v2.1 software.<br>- For proteomic library, data were searched independently using Pulsar in Spectronaut Professional+ v11.0.15038.<br>- Flow cytometry analyses were processed with FlowJo_V10.

- For karyotype analyses, data were analyzed using ChAS 3.2.
- Sanger sequencing electrophoregrams were analyzed on the Sequence Analysis Software V.5.2 (Applied Biosystems) and aligned and validated relative to the reference sequence using Sequencher V.5.4.6.

For manuscripts utilizing custom algorithms or software that are central to the research but not yet described in published literature, software must be made available to editors/reviewers. We strongly encourage code deposition in a community repository (e.g. GitHub). See the Nature Research guidelines for submitting code & software for further information.

Data

Policy information about availability of data

All manuscripts must include a data availability statement. This statement should provide the following information, where applicable:
- Accession codes, unique identifiers, or web links for publicly available datasets
- A list of figures that have associated raw data
- A description of any restrictions on data availability

The microarray datasets generated during and/or analysed during the current study are available in the GEO/NCBI (GEO accession: GSE108368, http://www.ncbi.nlm.nih.gov/geo/).
The mass spectrometry proteomics data that support the findings of this study have been deposited in the ProteomeXchange Consortium via the PRIDE partner repository with the dataset identifier PXD011652.
The mass spectrometry proteomics (Tandem Mass Tags - TMT) data have been deposited to the ProteomeXchange Consortium via the PRIDE partner repository with the dataset identifier PXD019316.

Databases used: Uniprot database (Swissprot entry only, release 2016_01, 16,747 entries) , CellAge database (http://genomics.senescence.info/cells/).

The authors declare that the data supporting the findings of this study are available within the paper and its supplementary information files. All the figures have associated source data.

No restriction is applied to the data presented.

Field-specific reporting

Please select the one below that is the best fit for your research. If you are not sure, read the appropriate sections before making your selection.

☒ Life sciences ☐ Behavioural & social sciences ☐ Ecological, evolutionary & environmental sciences

For a reference copy of the document with all sections, see nature.com/documents/nr-reporting-summary-flat.pdf

Life sciences study design

All studies must disclose on these points even when the disclosure is negative.

Sample size	No sample-size calculations were performed. Sample size was indicated in each figure legend and were determined based on the Authors' experience of what is necessary to generate a convincing and compelling result. Estimates were made based on our previous experience, experimental approach, availability and feasibility required to obtain statistically significant results. Sample size was determined to be adequate based on the magnitude and consistency of measurable differences between groups.
Data exclusions	Data were only excluded for failed experiments, like microbial contamination or obvious outliers.
Replication	All experiments were performed independently at least three times using biologically independent replicates, with essentially the same result. Data reported in this manuscript were reproduced over numerous independent experiments with sufficient cells/animals per group to demonstrate statistical significance. All replication attempts were successful. The findings in this paper were reproducible. The number of replicates are mentioned in the figure legends section.<br>Statistical analyses were performed with GraphPad Prism 6 software. Statistical significance was analyzed using two-tailed unpaired Student's t test between two different groups. For multiple comparisons, a one-way ANOVA analysis with Newman-Keuls post-test was performed. Results were considered significant when $P< 0.05$. Data are shown as mean ± SEM.
Randomization	For all the experiments performed, animals were assigned randomly to experimental and control groups. After sex and bodyweight randomization, animals were allocated in different groups.
Blinding	The investigators were not blinded during data collection or analysis, because most measurements performed in the paper were made using automated software, and not easily subject to operator bias. Also, data reported for mice experiments are quantitative, and not easily subject to operator bias.

Reporting for specific materials, systems and methods

We require information from authors about some types of materials, experimental systems and methods used in many studies. Here, indicate whether each material, system or method listed is relevant to your study. If you are not sure if a list item applies to your research, read the appropriate section before selecting a response.

Materials & experimental systems

n/a	Involved in the study	
☐	☒	Antibodies
☐	☒	Eukaryotic cell lines
☒	☐	Palaeontology
☐	☒	Animals and other organisms
☒	☐	Human research participants
☒	☐	Clinical data

Methods

n/a	Involved in the study	
☒	☐	ChIP-seq
☐	☒	Flow cytometry
☒	☐	MRI-based neuroimaging

Antibodies

Antibodies used

Smooth muscle alpha actin, α-SMA
- Supplier name: DAKO
- Clone: 1A4
- Lot: 20011632
- Catalog number: M0851
- Dilution: 1:50

Smooth muscle myosin heavy chain, SMMHC
- Supplier name: DAKO
- Clone: SMMS-1
- Lot: 10014006
- Catalog number: M355801-2
- Dilution: 1:50

Calponin 1/2/3
- Supplier name: Santa Cruz Biotechnology
- Clone: FL-297
- Lot: A1305
- Catalog number: sc-28545
- Dilution: 1:50

Lamin A/C
- Supplier name: Santa Cruz Biotechnology
- Clone: H-110
- Lot: D0412
- Catalog number: sc-20681
- Dilution: 1:50 for IF and 1:1000 for WB

Progerin
- Supplier name: Santa Cruz Biotechnology
- Clone: 13A4D4
- Lot: C1213
- Catalog number: sc-81611
- Dilution: 1:50 for IF and 1:1000 for WB

Osteopontin
- Supplier name: Santa Cruz Biotechnology
- Clone: AKm2A1
- Lot: A2513
- Catalog number: sc-21742
- Dilution: 1:50

Ki67
- Supplier name: DAKO
- Clone: MIB-1
- Lot: 00045312
- Catalog number: M7240
- Dilution: 1:50

H2AX (pS139)
- Supplier name: BD Biosciences
- Clone: N1-431 (RUO)
- Lot: 2349553
- Catalog number: 560443
- Dilution: 1:50

Heparan Sulfate
- Supplier name: United States Biological

- Clone: 8.S.087
- Lot: L14091230
- Catalog number: H1890
- Dilution: 1:263

Oct3/4
- Supplier name: Santa Cruz Biotechnology
- Clone: C-10
- Catalog number: sc-5279
- Lot: J2512
- Dilution: 1:50

Sox2
- Supplier name: Santa Cruz Biotechnology
- Clone: E-4
- Catalog number: sc-365823
- Lot: A2617
- Dilution: 1:50

CD31
- Supplier name: DAKO
- Clone: JC70A
- Catalog number: M0823
- Lot: 20055151
- Dilution: 1:50

p21
- Supplier name: Santa Cruz Biotechnology
- Clone: F-5
- Lot: E3018
- Catalog number: sc-6246
- Dilution: 1:50

Collagen type I
- Supplier name: Abcam
- Catalog number: ab34710
- Lot: GR3291638-2
- Dilution: 1:50

Fibronectin (EP5)
- Supplier name: Santa Cruz Biotechnology
- Clone: EP5
- Catalog number: sc-8422
- Lot: B0116
- Dilution: 1:50

Actin
- Supplier name: EMD Millipore Corp. USA
- Catalog number: MAB1501R
- Lot: 3137759
- Dilution: 1:5000

GAPDH
- Supplier name: EMD Millipore Corp. USA
- Catalog number: MAB374
- Lot: 2842113
- Dilution: 1:5000

VEGF Receptor 2, KDR (EPR21231)
- Supplier name: Abcam
- Catalog number: ab234110
- Lot: GR3230435-1
- Dilution: 1:20

CD34 - APC
- Supplier name: MACS Miltenyi Biotec
- Catalog number: 130-090-954
- Lot: 5161227192
- Dilution: 1:20

VeCad (F-8)
- Supplier name: Santa Cruz Biotech
- Catalog number: sc-9989
- Lot: D0317
- Dilution: 1:20

Anti-Mouse IgG Cy3
- Supplier name: Sigma
- Catalog number: C2181
- Dilution: 1:200

Cy3 AffiniPure Goat Anti Rabbit IgG
- Supplier name: JacksonImmunoResearch
- Catalog number: 111-165-144
- Lot: 118560
- Dilution: 1:100

Alexa Fluor 555 goat anti-mouse IgG
- Supplier name: Invitrogen
- Catalog number: A21422
- Lot: 1931963
- Dilution: 1:500

Alexa Fluor 488 donkey anti-mouse IgG
- Supplier name: Invitrogen
- Catalog number: A21202
- Lot: 2090565
- Dilution: 1:500

Alexa Fluor 488 goat anti-rabbit IgG
- Supplier name: Invitrogen
- Catalog number: A11034
- Lot: 2110499
- Dilution: 1:500

Validation

The antibodies are from commercial sources. For validation, the following methods were used: 1) in each immunofluorescence experiment, an isotype matched Ig control was used or incubation with the secondary antibody in the absence of the primary antibody and 2) manufacturer provided validation on the same species, relevant information on the antibodies are available on the manufacture's websites:

Smooth muscle alpha actin, α-SMA
- Site: https://www.agilent.com/store/productDetail.jsp?catalogId=M085101-2
- Validation: α-SMA Antibody (1A4), catalog number: M0851, DAKO, species: human origin by IHC

Smooth muscle myosin heavy chain, SMMHC
- Site: https://www.agilent.com/search/?Ntt=M355801-2&redirect=0
- Validation: SMMHC Antibody (SMMS-1), catalog number: M355801-2, DAKO, species: human origin by IHC

Calponin 1/2/3
- Site: https://www.scbt.com/p/calponin-1-2-3-antibody-fl-297?requestFrom=search
- Validation: Calponin 1/2/3 Antibody (FL-297), catalog number: sc-28545, Santa Cruz Biotechnology, species: Calponin 1 of mouse, rat and human origin, and Calponin 2 and Calponin 3 of human origin by WB, IP, IF, IHC(P) and ELISA

Lamin A/C
- Site: https://www.scbt.com/p/lamin-a-c-antibody-h-110?requestFrom=search
- Validation: Lamin A/C Antibody (H-110), catalog number: sc-20681, Santa Cruz Biotechnology, species: mouse, rat and human origin by WB, IP, IF, IHC(P) and ELISA; also reactive with additional species, including and equine, canine, bovine and porcine

Progerin
- Site: https://www.scbt.com/p/progerin-antibody-13a4d4?productCanUrl=progerin-antibody-13a4d4&_requestid=1537124
- Validation: Progerin antibody (13A4Da), catalog number: sc-81611, Santa Cruz Biotechnology, species: mouse, rat and human validated by WB and IP; non cross-reactive with Lamin A or Lamin C

Osteopontin
- Site: https://www.scbt.com/p/opn-antibody-akm2a1?requestFrom=search
- Validation: OPN Antibody (AKm2A1), catalog number: sc-21742, Santa Cruz Biotechnology, species: mouse, rat and human origin by WB, IP, IF and IHC(P)

Ki67
- Site: https://www.agilent.com/en/product/immunohistochemistry/antibodies-controls/primary-antibodies/ki-67-antigen-(concentrate)-76646
- Validation: Ki67 Antibody (MIB-1), catalog number: M7240, DAKO, species: human origin by IHC and WB

H2AX (pS139)
- Site: https://www.bdbiosciences.com/us/applications/research/apoptosis/purified-antibodies/purified-mouse-anti-h2ax-ps139-n1-431/p/560443
- Validation: H2AX (pS139), catalog number: 560443, BD Biosciences, species: human (QC Testing), mouse (Tested in Development) origin by flow cytometry (Routinely Tested), bioimaging and WB (Tested During Development)

Heparan Sulfate
- Site: https://www.usbio.net/antibodies/H1890/heparan-sulfate-10e4-epitope
- Validation: Heparan Sulfate (10E4), catalog number: H1890, US Biological, species: human origin by Flow Cytometry, IHC, ELISA and WB

Oct3/4
- Site: https://www.scbt.com/p/oct-3-4-antibody-c-10?requestFrom=search
- Validation: Oct-3/4 Antibody (C-10), catalog number: sc-5279, Santa Cruz Biotechnology, species: mouse, rat and human origin by WB, IP, IF, IHC(P), FCM and ELISA; non cross-reactive with Oct-3/4 isoform B

Sox2
- Site: https://www.scbt.com/p/sox-2-antibody-e-4?requestFrom=search
- Validation: Sox-2 Antibody (E-4), catalog number: sc-365823, Santa Cruz Biotechnology, species: mouse, rat and human origin by WB, IP, IF, IHC(P) and ELISA; also reactive with additional species, including and equine, canine and bovine

CD31
- Site: https://www.agilent.com/cs/library/packageinsert/public/SSM0823CEEFG_01.pdf
- Validation: CD31 Antibody (JC70A), catalog number: M0823, DAKO, species: human origin by IHC

p21
- Site: https://www.scbt.com/p/p21-antibody-f-5?requestFrom=search
- Validation: p21 Antibody (F-5), catalog number: sc-6246, Santa Cruz Biotechnology, species: mouse, rat and human origin by WB, IP, IF, IHC(P) and FCM

Collagen type I
- Site: https://www.abcam.com/collagen-i-antibody-ab34710.html
- Validation: Collagen type I, catalog number: ab34710, Abcam, species: Mouse, Rat, Sheep, Goat, Horse, Cow, Human, Pig, Common marmoset origin by IHC, WB, ELISA, ICC/IF, IP

Fibronectin (EP5)
- Site: https://www.scbt.com/p/fibronectin-antibody-ep5?requestFrom=search
- Validation: Fibronectin Antibody (EP5), catalog number: sc-8422, Santa Cruz Biotechnology, species: mouse, rat and human origin by WB, IP, IF, IHC(P) and ELISA

Actin
- Site: https://www.merckmillipore.com/PT/en/product/Anti-Actin-Antibodyclone-C4,MM_NF-MAB1501R?ReferrerURL=https%3A%2F%2Fwww.google.com%2F
- Validation: Actin, catalog number: MAB1501R, EMD Millipore Corp, species: A origin by ELISA, ICC, IHC, IH(P), WB

GAPDH
- Site: https://www.merckmillipore.com/PT/en/product/Anti-Glyceraldehyde-3-Phosphate-Dehydrogenase-Antibody-clone-6C5,MM_NF-MAB374
- Validation: GAPDH, catalog number: MAB374, EMD Millipore Corp, species: Ca, H, M, R, Rb, F, Fe, Po origin by ELISA, IP, ICC, IF, IHC, WB

VEGF Receptor 2, KDR (EPR21231)
- Site: https://www.abcam.com/vegf-receptor-2-antibody-epr21231-ab234110.html
- Validation: KDR (EPR21231), catalog number: ab234110, Abcam, species: human origin by ICC/IF, Flow Cytometry

CD34 - APC
- Site: https://www.citeab.com/antibodies/2089642-130-090-954-cd34-apc-human-monoclonal
- Validation:CD-34-APC, catalog number: 130-090-954, MACS Miltenyi Biotec, species: human origin by FC/FACS

VeCad (F-8)
- Site: https://www.scbt.com/p/ve-cadherin-antibody-f-8?requestFrom=search
- Validation: VE-cadherin Antibody (F-8), catalog number: sc-9989, Santa Cruz Biotechnology, species: mouse, rat and human origin by WB, IP, IF, FCM and ELISA; also reactive with additional species, including porcine

Anti-Mouse IgG Cy3
- Site: https://www.sigmaaldrich.com/catalog/product/sigma/c2181?lang=pt&region=PT
- Validation: Anti-Mouse IgG Cy3, catalog number: C2181, Sigma, species: mouse origin by IHC

Cy3 AffiniPure Goat Anti Rabbit IgG
- Site: https://www.jacksonimmuno.com/catalog/products/111-165-144
- Validation: Anti-Rabbit IgG Cy3, catalog number: 111-165-144, JacksonImmunoResearch, species: rabbit origin

Alexa Fluor 555 goat anti-mouse IgG
- Site: https://www.thermofisher.com/antibody/product/Goat-anti-Mouse-IgG-H-L-Cross-Adsorbed-Secondary-Antibody-Polyclonal/A-21422
- Validation: Alexa Fluor 555 goat anti-mouse IgG, catalog number: A21422, Invitrogen, species: mouse origin by ICC, IF, IHC, Flow cytometry and WB

Alexa Fluor 488 donkey anti-mouse IgG
- Site: https://www.thermofisher.com/antibody/product/Donkey-anti-Mouse-IgG-H-L-Highly-Cross-Adsorbed-Secondary-Antibody-Polyclonal/A-21202

- Validation: Alexa Fluor 488 donkey anti-mouse IgG, catalog number: A21202, Invitrogen, species: mouse origin by ICC, IF, IHC and Flow cytometry

Alexa Fluor 488 goat anti-rabbit IgG
- Site: https://www.thermofisher.com/antibody/product/Goat-anti-Rabbit-IgG-H-L-Highly-Cross-Adsorbed-Secondary-Antibody-Polyclonal/A-11034
- Validation: Alexa Fluor 488 goat anti-rabbit IgG, catalog number: A11034, Invitrogen, species: rabbit origin by ICC, IF, IHC and Flow cytometry

Eukaryotic cell lines

Policy information about cell lines

Cell line source(s)	HGPS fibroblasts line AG06917 (Coriell cell repositories); hvSMCs (human vascular smooth muscle cells) (Lonza, CC-2579); HaCaT (human immortalized keratinocyte cell line) (Lonza); MEFs (mouse embryonic fibroblasts) (GlobalStem, GSC-6001M); N-iPSCs (k2 iPSCs line generated by Ulrich Martin Lab); HGPS-iPSCs (iPSCs line generated by Xavier Nissan Lab); HUAECs (human umbilical artery endothelial cells) (Cell Applications, inc, 095202-05n)
Authentication	Some of these cells were commercially available cell lines, so no further cell line authentication was performed. For iPSCs, microscopic inspection was performed (these cells are easily distinguished based on morphology), as well as immunofluorescence for some pluripotent markers.
Mycoplasma contamination	The cell lines were tested and are free from mycoplasma contamination.
Commonly misidentified lines (See ICLAC register)	No commonly misidentified cell lines were used in the study.

Animals and other organisms

Policy information about studies involving animals; ARRIVE guidelines recommended for reporting animal research

Laboratory animals	Details on the mice used in this study have been included in the Methods section of this manuscript.<br>Male and female (C57BL/6 background) LmnaG609G/G609G, LmnaG609G/+, Mmp13-/+ and wild-type mice were used. 6 or 10 or 18-week old mice were used in this study. Mice were kept in cages in standard housing conditions (temperature, 22ºC± 2ºC; humidity, 55% ± 5%; 12-hour light/ 12-hour dark cycle).
Wild animals	No wild animals were used in the study.
Field-collected samples	No field collected samples were used in the study.
Ethics oversight	Animal studies were approved by the local ethical committee (Marseille Animal Care Commitee, Protocol n° 96-21122012) and were in accordance with the Directive 2010/63/EU of the European parliament regarding the protection of animals used for experimental and other scientific purposes.<br>Procedures were approved by the Ethics Committee of Animal Experimentation (CCEA 57/16) of the Vall d'Hebron Research Institute and were conducted in compliance with Spanish legislation and in accordance with the Directives of the European Union.

Note that full information on the approval of the study protocol must also be provided in the manuscript.

Flow Cytometry

Plots

Confirm that:

☒ The axis labels state the marker and fluorochrome used (e.g. CD4-FITC).

☒ The axis scales are clearly visible. Include numbers along axes only for bottom left plot of group (a 'group' is an analysis of identical markers).

☒ All plots are contour plots with outliers or pseudocolor plots.

☒ A numerical value for number of cells or percentage (with statistics) is provided.

Methodology

Sample preparation	Cells were dissociated with non-enzymatic cell dissociation buffer (Gibco) for 10 min, followed by gentle pipetting and washes in PBS with 5% (v/v) FBS. Single cells were aliquoted in PBS with 5% FBS (200,000 cells were used per condition) and stained with either isotype controls or antigen-specific fluorescent-conjugated antibodies (CD34) for 30 min at 4ºC. For VE-Cadherin (F-8, Santa Cruz Biotech) and KDR (anti-VEGF receptor 2 antibody [EPR21231] (Abcam) analysis cells were fixed with 1% paraformaldehyde (PFA) for 10 min, permeabilized with triton 0.1% for 10 min, and were incubated with primary antibody for 1 h at room temperature followed by the secondary antibody for 30 min at room temperature.
Instrument	The flow cytometry analyses were performed in a BD Accuri C6, BD Biosciences.

Software	Analyses were processed with FlowJo_V10.
Cell population abundance	At least ten thousand events were collected. The percentages showed in the histograms were calculated based on the isotype controls represented by orange (the gate for the isotype was defined at 1%).
Gating strategy	The gate was defined for the isotype at 1%

☒ Tick this box to confirm that a figure exemplifying the gating strategy is provided in the Supplementary Information.
